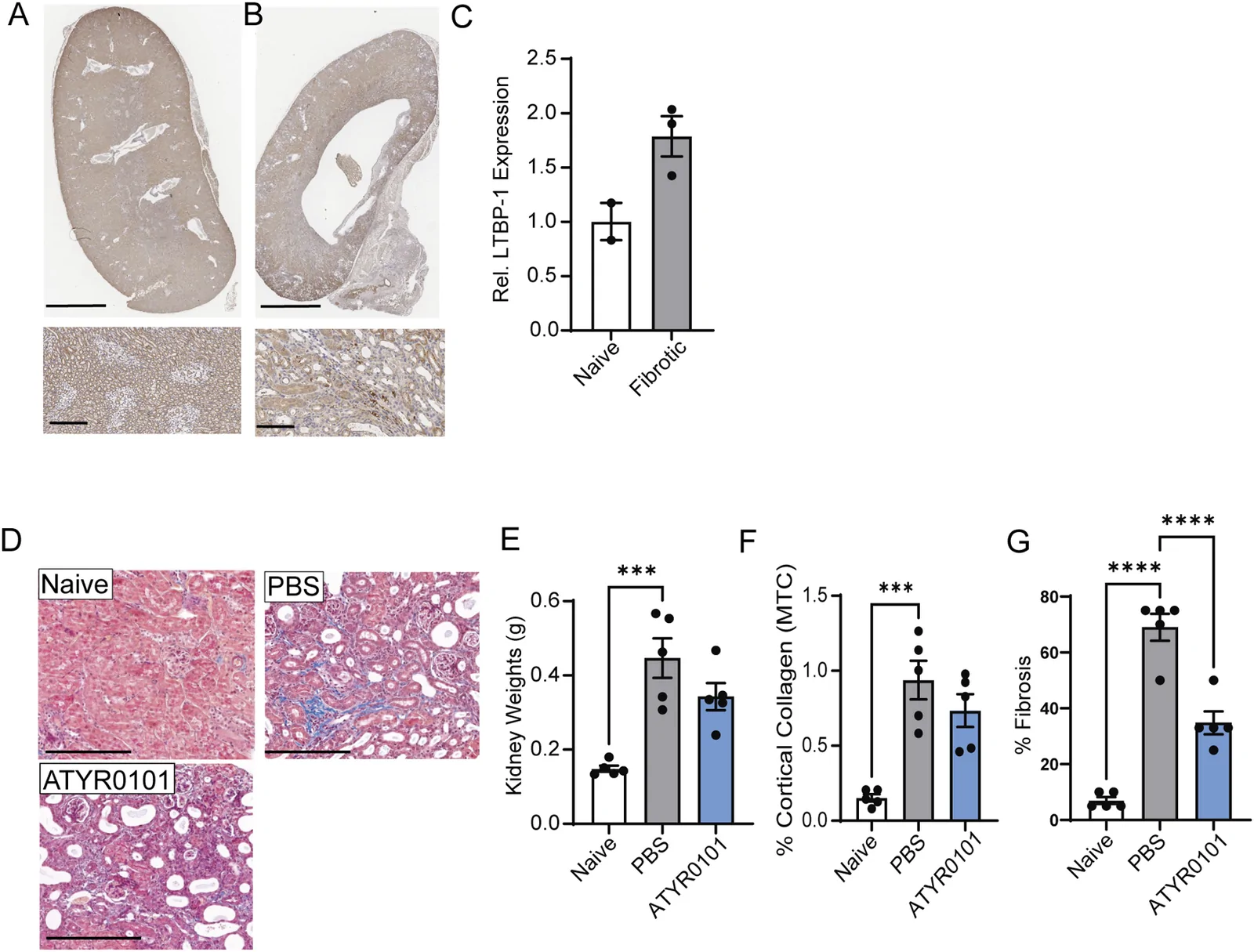# Natural Asp-tRNA synthetase fragment targets myofibroblasts to abrogate tissue fibrosis

**DOI:** 10.1016/j.jbc.2026.113586

**Published:** 2026-09-30

**Authors:** Ryan A. Adams, Ying-Ting Wang, Alison G. Barber, Kristina Hamel, Andrew Imfeld, Luke Burman, Kaitlyn Rauch, Yeeting E. Chong, Angelica Villanueva, Lauren Guy, Wayne Liu, Crystal Manzo, Sofia Klopp-Savino, Clara Polizzi, Jasmine Stamps, Max Pastenes, Honglei Tian, Chun Po Fung, Yingyi Zhang, Kwok Wa Hugo Ho, Bruno Sangiorgi, Blythe Dillingham, Tarsis F. Brust, Kyle P. Chiang, Zhenguo Wu, Zhiwen Xu, Christoph Burkart, Leslie A. Nangle

**Affiliations:** 1 aTyr Pharma, San Diego, California, USA; 2 IAS HKUST R&D Laboratory, Institute for Advanced Study, Hong Kong University of Science and Technology, Kowloon, Hong Kong, China; 3 Division of Life Science, The Hong Kong University of Science and Technology, Hong Kong, China; 4 Pangu Biopharma, Hong Kong, China; 5 Biological Cryo-EM Center, The Hong Kong University of Science and Technology, Hong Kong, China; 6 Alimentiv Inc, London, Ontario, Canada

**Keywords:** fibrosis, myofibroblasts, apoptosis, FAK, aminoacyl tRNA synthetase, mitochondria, anoikis

## Abstract

Fibrosis remains a major global health challenge with limited therapies that only slow disease progression. While the aminoacyl-tRNA synthetase (aaRS) family is known to utilize evolutionarily appended domains as extracellular signaling molecules, their role in regulating the homeostasis of the fibrotic process is poorly defined. We identify a natural proteolytic fragment of aspartyl-tRNA synthetase (DARS), termed DARS-N1, that selectively triggers apoptosis in myofibroblasts by targeting latent-transforming growth factor beta binding protein 1 (LTBP-1) on the extracellular matrix (ECM). This active N-terminal fragment of DARS reduces focal adhesion kinase (FAK) activation in undifferentiated fibroblasts and myofibroblasts, while only myofibroblasts exhibit inadequate cell-matrix attachment leading to loss of anchorage-dependent survival and promotion of apoptosis. We utilized ATYR0101, a therapeutic form of DARS-N1 engineered for extended serum exposure, to demonstrate anti-fibrotic activity in lung (bleomycin). Spatial transcriptomics analysis showed that ATYR0101 activates a restorative program promoting the return to a healthy state. These findings highlight ATYR0101’s potential as a transformative therapy for disrupting the process of chronic tissue fibrosis.

Tissue injury triggered by genetic, environmental or inflammatory insults can lead to tissue fibrosis, a systemic pathology affecting multiple organs, including lung, liver, kidney, heart, and skin (1). Common fibrosis-related diseases such as interstitial lung disease, idiopathic pulmonary fibrosis (IPF), cystic fibrosis, viral hepatitis, non-alcoholic steatohepatitis, cirrhosis, chronic renal disease, heart failure, and scleroderma demonstrate an average annual incidence of approximately 5000 per 100,000 person-years, causing a high impact on morbidity and mortality rates worldwide (2). An estimated 45% of all deaths in the United States are attributable to severe fibrotic disorders (3) characterized by excess extracellular matrix (ECM) deposition that distorts tissue architecture and leads to organ failure. Central to this process is the activation of fibroblasts to myofibroblasts, which synthesize and remodel the ECM, directing the deposition of collagen and expression of α-smooth muscle actin, thereby enhancing contractile activity (4). While physiological repair is transient, pathological fibrosis is driven by the evasion and persistence of myofibroblasts (5). The persistence of activated myofibroblasts is reinforced by biochemical signals (*e.g.*, cytokines, growth factors and ECM ligands), in particular TGF-β, and mechanical factors (*e.g.*, matrix stiffness). Transient stiffening of ECM plays a crucial role in protecting wounds from rupture, whereas increased matrix stiffness in the fibrotic ECM resulting from excessive collagen deposition and aberrant, intensified myofibroblast contraction sustains activation and promotes the prolonged persistence of myofibroblasts, thus creating a self-perpetuating feedback cycle of fibrosis (5). Consequently, targeting myofibroblast cell fate to trigger apoptosis represents a promising therapeutic strategy for resolving established fibrotic disease.

In parallel with advances in understanding the cellular mechanisms driving fibrosis, recent years have revealed an unexpected role for aminoacyl-tRNA synthetases (aaRSs) beyond their canonical functions in protein synthesis (6, 7). Traditionally recognized as essential enzymes catalyzing the attachment of amino acids to their corresponding tRNAs, aaRSs are now known to exert diverse extracellular and non-canonical functions that influence key physiological and pathological processes. These include the regulation of angiogenesis, immune and inflammatory signaling, transcriptional control, oncogenesis, and neurological function (8, 9, 10, 11, 12, 13, 14). The emergence of these novel activities correlates with the evolutionary acquisition of additional, non-catalytic domains unique to higher eukaryotes. Once incorporated, these domains have been conserved, suggesting adaptive value and specialized roles in intercellular communication and stress responses (15, 16). Liberation of these newly evolved domains from the catalytic core can occur through two primary mechanisms: alternative splicing and proteolytic cleavage (17). Both processes rely on unconventional secretion pathways, either transporting the variant or full-length enzyme to the extracellular space. In the latter case, activation of the newly evolved domains is mediated by tissue-specific or pathology-specific proteases, enabling them to bind extracellular targets and mediate signaling. Examples of this include cleavage of Tyr-tRNA synthetase by elastase and matrix metalloproteinases (18) and His-tRNA synthetase by Granzyme B (19).

The existence of truncated variants of aaRS is consistent with a growing understanding *via* various proteomics efforts and large-scale analyses of the existence of a deeper complexity in the functioning proteome that exists beyond the approximately 20,000 proteins encoded by traditional open reading frames (ORFs) (20, 21, 22). Known as the “dark proteome”, these non-canonical proteins generated by methods including alternative splicing, proteolysis and use of non-traditional ORFs are present in addition to the approximately 20,000 proteins encoded by traditional open reading frames and represent a largely uncharted reservoir of potential new signaling molecules and pathways (20, 21, 22).

In this study, we sought to characterize the nature of aaRS-related non-canonical proteins regulated under inflammatory conditions utilizing proteomics mass spectrometry and ultimately define their extracellular targets, downstream functional effects and potential therapeutic utility. Interestingly, one of the proteins identified to be regulated during inflammation both *in vitro* and *in vivo* was an extracellular N-terminal fragment of DARS (DARS-N1) that encompasses its unique helical domain, which is connected to the strictly conserved DARS catalytic domain by a structurally disordered flexible linker with apparent accessibility to proteases. This newly evolved domain appears in organisms as early as vertebrates, a similar point in evolution where fibrotic processes are first seen (23). We conducted target identification studies that revealed the extracellular target of this domain to be latent transforming growth factor beta binding protein 1 (LTBP-1). LTBP-1 acts as an extracellular matrix protein that regulates the availability of bioactive TGF-β and downstream TGF-β signaling (24). A therapeutic form based on this natural proteolytic fragment conjugated with a modified IgG4 (ATYR0101) was engineered to confer extended serum exposure and maintain molecular homogeneity to facilitate functional studies. Due to LTBP-1s connections to key aspects of immune signaling and fibrosis, efforts focused on elucidating a potential role for the DARS-N1 fragment in modulating these essential processes and investigating its potential application as an anti-fibrotic therapeutic.

## Results

### Natural proteolytic DARS fragment is liberated under inflammatory conditions and binds LTBP-1

To explore the multi-functionality of tRNA synthetases under inflammatory conditions and to identify naturally occurring protein fragments, we characterized the secreted proteome from activated human macrophages (THP-1 cells), key cells in both the initiation and resolution of inflammation, using LC/MS/MS proteomic analysis. Macrophages represent a physiologically relevant cell type in tissue inflammation, but this process of tRNA synthetase secretion could occur in many cell types. In this approach, we detected a protein fragment of cytosolic DARS that comprises the N-terminal helix and anticodon-binding domain, designated DARS-N1, encompassing human residues 1-154 (Table S1). This DARS-N1 fragment was only observed in the extracellular space and a result of stimulation *via* LPS treatment (Fig. 1*A*). Western blot analysis confirmed that the observed 20 kDa fragment represented human DARS-N1 (the immunoreactive band at ∼30 kDa was excluded as a DARS-derivative based on the absence of DARS-specific peptides in the LC/MS/MS fractions corresponding to 25-40 kDa). Mass spectrometry also confirmed the presence of the full-length (FL) human DARS in the media of differentiated THP-1 monocytes after activation with LPS, thus suggesting DARS-N1 arises from extracellular proteolytic cleavage of FL DARS. To further probe the specificity of this cleavage site, we examined the potential to liberate this domain with endogenous proteases. We treated recombinant human DARS with PMN elastase and observed clear evidence (by gel electrophoresis and LC/MS/MS analysis) of the release of DARS-N1 by cleavage after Leucine 154 (Fig. S1). A fragment consistent with DARS-N1 was also generated by *in vitro* digestion with MMP-12.

**Figure 1:**
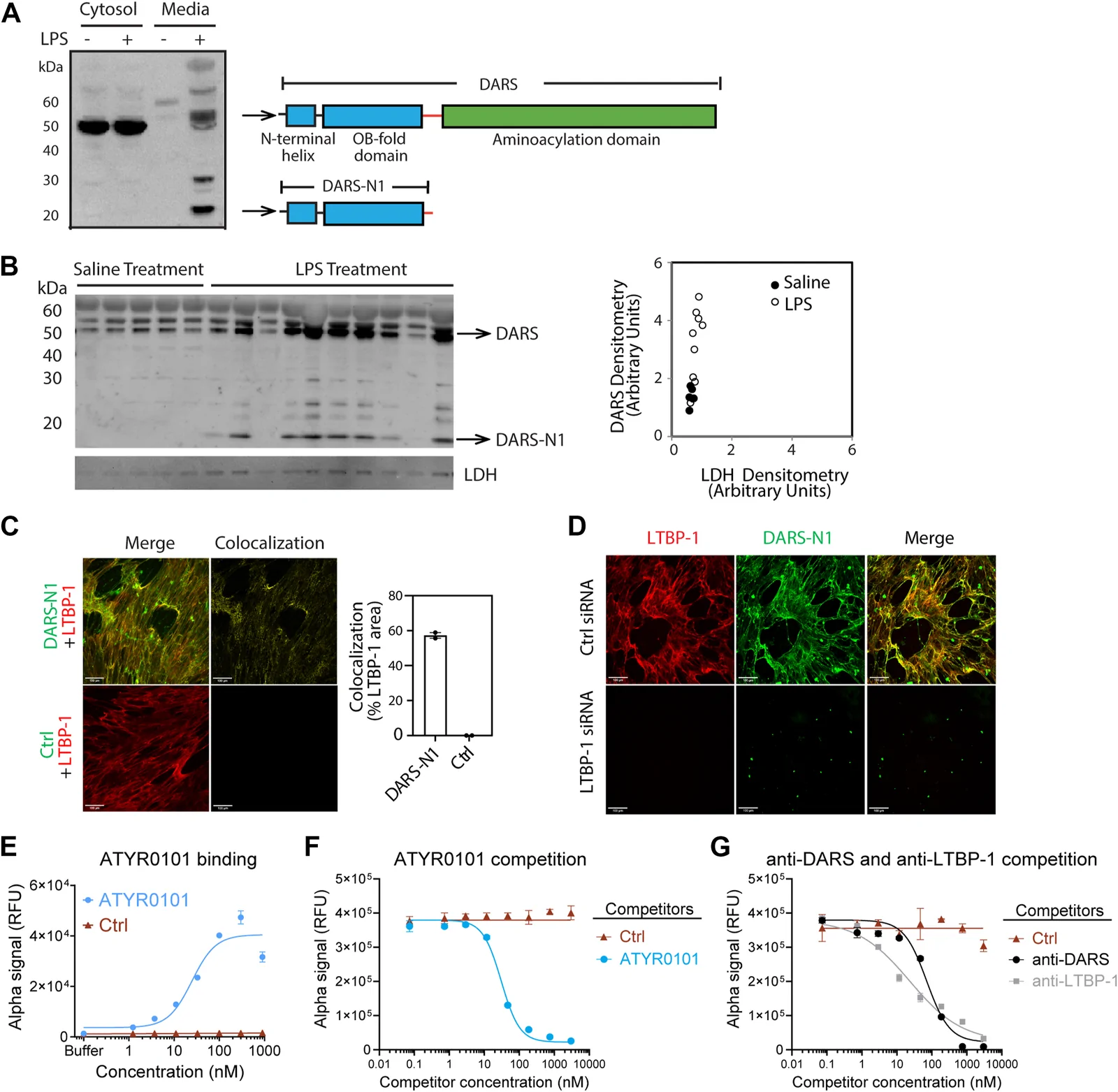
**An N-terminal fragment of DARS released from activated macrophages binds to LTBP-1.***A*, THP-1 human macrophages were stimulated with LPS and the resulting conditioned media and cytosol were subjected to SDS-PAGE analysis and immunoblotted with an anti-DARS polyclonal antibody. Gel bands (20 in total from 75-10 kDa) from conditioned media of LPS treated macrophages were subjected to in-gel trypsin digestion and analyzed by LC/MS/MS. The anti-DARS reactive band at ∼50 kDa was identified as full length human DARS while the band at ∼20 kDa corresponded to an N-terminal fragment of DARS termed DARS-N1. *B*, bronchoalveolar lavage fluid (BALF) samples from saline or LPS treated mice were probed for FL DARS and fragments. Lactate dehydrogenase (LDH) presence in BALF samples was assessed using an anti-LDH antibody. Densitometry analysis was performed and intensities of FL DARS bands were plotted against LDH bands. *C*, WI-38 cells were treated with TGF-β to induce extracellular matrix deposition. After 6 days, cells were removed, and then ECM were stained with DARS-N1 or a control protein (non-binding synthetase fragment from methionyl-tRNA synthetase) in *green* and LTBP-1 in *red*. Quantification of (*C*) as a percentage of the LTBP-1 staining area. Scale bars are 100 μm. *D*, TGF-β-differentiated WI-38 cells were treated with control siRNA (*top*) or LTBP-1 siRNA (*bottom panel*). ECM from control or LTBP-1 knockdown cells were stained with DARS-N1 or a control protein (non-binding synthetase fragment from Methionyl-tRNA synthetase) in *green*, and LTBP-1 in *red*. Scale bars are 100 μm. *E*, direct protein–protein interaction between ATYR0101 and recombinant human LTBP-1 was shown by AlphaLISA. *F* and *G*, ATYR0101 competitive binding assay to measure the AlphaLISA signal upon exogenous competitor, (*F*) ATYR0101 and human IgG4 Fc control protein, (*G*) anti-DARS antibodies, anti-LTBP-1 antibodies or anti-IgG control antibodies, in competition with the binding of ATYR0101 (donor beads) to LTBP-1 (acceptor beads). Data are shown as mean ± SEM. n = 3 independent studies.

To examine potential *in vivo* production of DARS-N1, we examined its presence in the context of acute inflammation in the mouse. Intranasal LPS application induces acute lung inflammation driven to a great extent by neutrophil and macrophage invasion into the lung tissue and secretion of their active proteases (PMN elastase and MMP-12, respectively) into the bronchoalveolar lavage fluid (BALF). Immunoblotting of BALF samples, after 2 days of intranasal LPS administration, with α-DARS polyclonal antibodies demonstrated a significant increase in extracellular FL DARS when compared to BALF from saline treated animals (Fig. 1*B*). Levels of FL DARS immunoreactivity correlated with increased cell infiltration of primarily neutrophils and macrophages. This increase was accompanied in several animals with the appearance of smaller fragments of DARS consistent in size with those confirmed to be DARS-N1. Levels of another cytosolic protein, lactate dehydrogenase (LDH), were not observed to accumulate significantly in the BALF following intranasal LPS administration (Fig. 1*B*). These observations showed that the appearance of extracellular FL DARS and DARS-N1 cannot be attributed solely to overall cell breakdown and instead are released by cells in response to an acute inflammatory event.

To identify the target for DARS-N1, we employed a cross-linking ligand-receptor capture assay followed by purification and liquid chromatography-mass spectrometry called TriCEPS technology by Dualsystems Biotech. LTBP-1 was identified as a binding partner of DARS-N1 (Fig. S2*A*). LTBP-1 is an ECM protein and serves as a key modulator of TGF-β signaling. Next, we validated the interaction between DARS-N1 and LTBP-1 in the ECM. Immunofluorescence analysis revealed that DARS-N1 bound to ECM deposited by TGF-β-stimulated human pulmonary fibroblast WI-38 cells and colocalized with LTBP-1 (Figs. 1*C* and S2*B*). Additionally, siRNA knockdown of LTBP-1 in TGF-β-differentiated myofibroblasts abrogated the binding of DARS-N1 onto the ECM, further confirming the direct binding between LTBP-1 and DARS-N1 (Fig. 1*D*).

### Engineering of ATYR0101, a stable therapeutic DARS-N1-Fc fusion that selectively binds LTBP-1

Due to DARS-N1's relatively short serum half-life in the mouse (Fig. S3*B*) and the need to generate a therapeutically viable molecule for murine *in vivo* studies, we engineered ATYR0101 by fusing DARS-N1 (residue 3-154) with the Fc region of human IgG4 containing an S228P and L235E mutations to mitigate Fc effector function (25). Three mutations (R81C, N86C and C130S) in DARS-N1 were also introduced. These mutations including disulfide bridges R81C/N86C combined with the mutation C130S were designed using Disulfide by Design 2.0 to improve ATYR0101 stability (26, 27). The protein was expressed in Expi293F cells, purified with protein A chromatography, and further confirmed to form a dimer by non-reduced and reduced SDS-PAGE. An ATYR0101 modeled structure was designed to illustrate the final molecule (Fig. S3*A*) and demonstrated an improvement in serum exposure (Fig. S3*B*) and molecular homogeneity (Fig. S3*C*) relative to its parental DARS-N1 fragment.

To better understand the pharmacological relationship between ATYR0101 and its target LTBP-1, we demonstrated direct protein-protein interaction between ATYR0101 and LTBP-1 using AlphaLISA, resulting in a median binding effective concentration (EC_50_) of 26 nM (Fig. 1*E*). This interaction was selectively inhibited by the addition of exogenous competitors ATYR0101, anti-DARS antibodies and anti-LTBP-1 antibodies with half-maximal inhibitory concentration (IC_50_) of 30.6 nM, 71.4 nM, and 23.4 nM, respectively (Fig. 1, *F* and *G*). To further identify the minimal binding domain required, we expressed human LTBP-1 short form (LTBP-1S) along with truncated LTBP-1 fragments containing cysteine-rich domains (Fig. S4*A*). We performed ATYR0101 AlphaLISA binding to validate their interaction and demonstrated direct binding between ATYR0101 and LTBP-1S and two LTBP-1 fragments, L1S_1006-1395 and L1S_1186-1395 indicating that ATYR0101 targets the C-terminal region of LTBP-1 (Fig. S4, *B* and *C*). Furthermore, ATYR0101 confirmed a broad species specificity with similar binding EC_50_ observed across distinct species LTBP-1 tested, including human, mouse, rat and cynomolgus monkey (Fig. S4*D*). Altogether, these findings confirm LTBP-1 as the binding partner of ATYR0101.

### ATYR0101 induces selective apoptosis in myofibroblasts while sparing undifferentiated fibroblasts

ATYR0101 specifically targets LTBP-1, an extracellular reservoir of latent TGF-β, which is recognized as a major driver of tissue fibrosis. To investigate the potential utility for ATYR0101 in fibrotic pathologies, we first assessed the morphology changes of human lung fibroblasts (WI-38 cells) or TGF-β-differentiated WI-38 myofibroblasts with ATYR0101 treatment. No observable difference was found in cell morphology or the total cell confluency between ATYR0101-treated and control-treated undifferentiated fibroblasts (Fig. 2*A*, left panel). Notably, ATYR0101 treated TGF-β-differentiated myofibroblasts exhibited a distinct apoptotic morphological appearance, such as abrupt cell shrinkage, cell aggregation and eventual cell loss, compared with control-treated myofibroblasts (Fig. 2*A*, right panel). Increased caspase-3/7 levels in TGF-β-differentiated myofibroblasts upon ATYR0101 treatment were further confirmed by real-time kinetic live cell imaging (Fig. 2*A*). In addition, a luminescent assay measuring cleaved caspase-3/7 was used to confirm ATYR0101 dose-dependent induction of myofibroblast apoptosis. The EC_50_ to induce myofibroblast apoptosis was found to be 190 nM (Fig. 2*B*). ATYR0101 was consistently found to induce apoptosis selectively in TGF-β-differentiated myofibroblasts while having no effect on undifferentiated fibroblasts, as shown in other pulmonary fibroblast cell lines (MRC-5) as well as primary lung fibroblasts from both idiopathic pulmonary fibrosis (IPF) patients and healthy donors (NHLF) (Fig. 2, *C* and *D*). While ATYR0101 induced caspase-3/7 mediated apoptosis selectively in TGF-β-differentiated myofibroblasts, ATYR0101 showed a negligible effect on lung epithelial cells (A549) or TGF-β induced epithelial-to-mesenchymal transition of A549 cells (Fig. S5).

**Figure 2:**
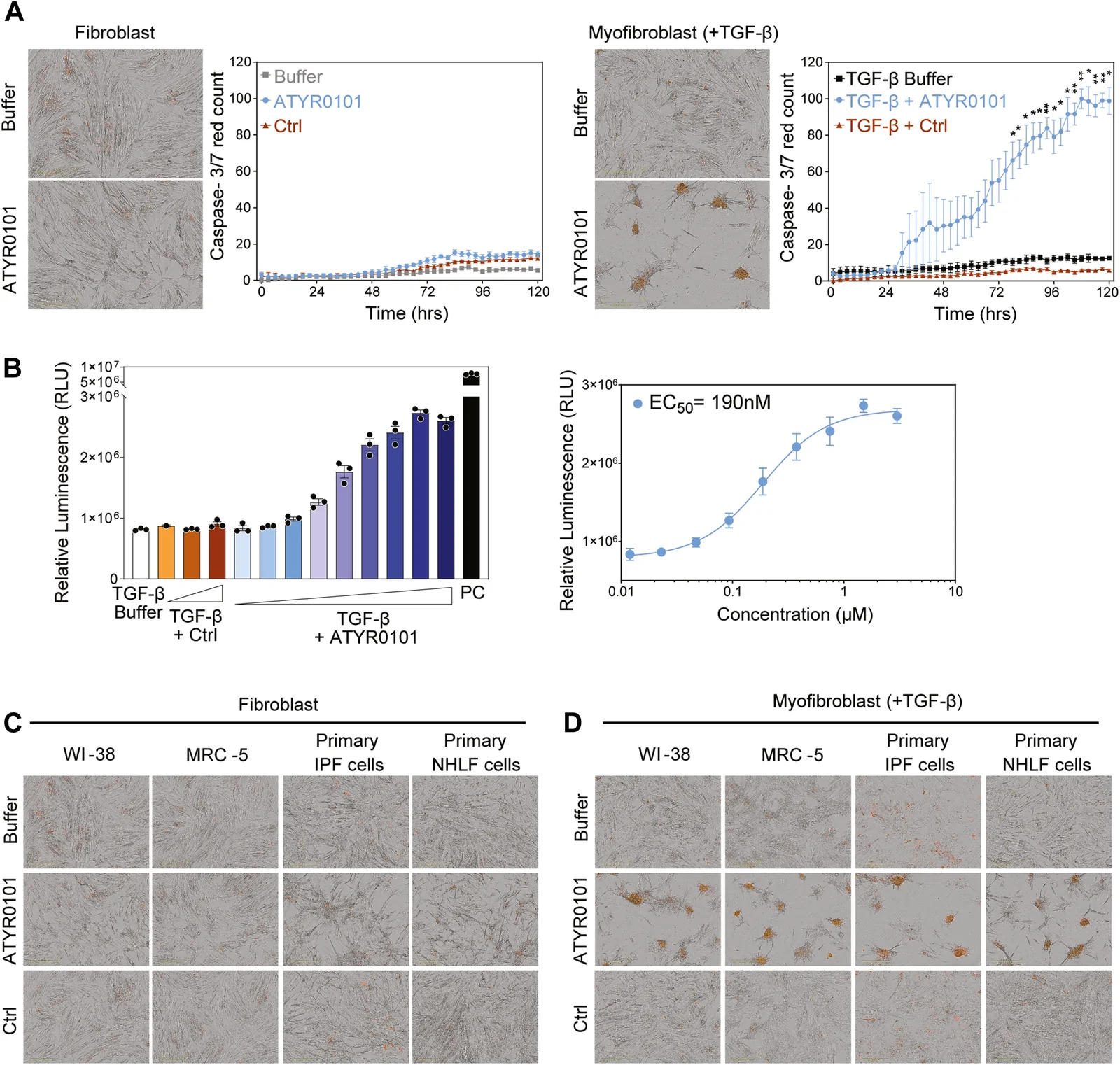
**ATYR0101 induces TGF-β-differentiated myofibroblast apoptosis.***A*, representative images are shown for WI-38 fibroblasts treated with assay buffer (*left*) or with TGF-β (*right*) in the presence of ATYR0101 (600 nM) or human IgG4 Fc control protein and stained for activated caspase-3/7. Kinetic plot of caspase-3/7 positive signals during 5 days of treatment quantified through live-cell imaging. Three independent experiments were performed. Each sample was run in triplicate. Scale bars are 400 μm. *B*, representative data showing dose-dependent responses of ATYR0101 for myofibroblast apoptosis. WI-38 myofibroblasts were treated with ATYR0101 or a human IgG4 Fc control protein starting at 3 μM with a 2-fold serial dilution or TGF-β alone for 2-3 days. Staurosporine (5 μM) serves as positive control (PC). Caspase-3/7 activities measuring luminescent signals were quantified using Caspase-Glo 3/7 Assay (n = 5 independent studies. *C* and *D*, representative images of Buffer alone (*top*) or in combination with ATYR0101 (*middle*) or human IgG4 Fc control protein (*bottom*)-treated pulmonary fibroblasts (*C*) or TGF-β-differentiated myofibroblasts (*D*) from cell lines (WI-38 and MRC-5) and primary cells (originated from IPF patients and normal healthy donors) for 3-4 days and stained for annexin V activity. n = 3 independent studies for each cell line. Data are shown as mean ± SEM. n = 3 independent studies. ∗*p* < 0.05, ∗∗*p* < 0.01, and ∗∗∗*p* < 0.001 by two-way ANOVA followed by Tukey’s multiple group comparisons test. Scale bars are 400 μm.

Notably, TGF-β treated WI-38 cells in the presence of ATYR0101 exhibited a lack of cell attachment at early time points compared to TGF-β buffer alone controls, further correlating with ATYR0101 induced myofibroblast apoptosis (Fig. 3*A*). To determine whether ATYR0101 inhibited anchorage-dependent cell survival, potentially promoting anoikis—a type of programmed cell death (apoptosis) specifically induced by inadequate cell-matrix interactions, we assessed the phosphorylation of focal adhesion kinase (FAK) in WI-38 cells co-treated with ATYR0101 and TGF-β. The dose-dependent attenuation of FAK phosphorylation at Tyr497 found in ATYR0101 treated TGF-β differentiated WI-38 myofibroblasts was confirmed by ELISA (Fig. 3*B*) and Western blot analysis (Fig. 3*C*). In addition, we analyzed the focal adhesions of pre-attached fibroblast followed by ATYR0101 or control protein treatment in the presence of TGF-β. Dual-immunoflurescent analysis of focal adhesions using antibodies targeting total FAK and phosphorylated FAK (Tyr397) demonstrated that ATYR0101 treatment led to a diffused cytosolic total FAK protein distribution compared to ctrl treated myofibroblast. Furthermore, a decreased FAK phosphorylation and reduced colocalization with in the focal adhesions of WI-38 myofibroblasts was found in ATYR0101 treated myofibroblast (Fig. 3*D*). Inhibition of FAK activation in myofibroblasts induced by the interaction of ATYR0101 with LTBP-1 may disrupt the mechanical or biochemical scaffolding required for myofibroblast anchorage leading to a loss of cell attachment during differentiation and ultimately apoptosis. Since mitochondria play a critical role in apoptosis induction, we next asked whether mitochondrial health was compromised in ATYR0101 treated myofibroblasts. TGF-β-differentiated WI-38 myofibroblasts were stained with JC-1 dye, a membrane-permeant indicator of mitochondria membrane potential. ATYR0101 treated myofibroblasts exhibited a significant increase in mitochondria depolarization, as indicated by enhanced green fluorescence signals of JC-1 monomers compared to control treatments (Fig. 3*E*). Overall, ATYR0101 treatment reduces FAK phosphorylation and subsequently induces mitochondrial depolarization followed by caspase-3/7 mediated apoptosis exclusively within TGF-β-differentiated pulmonary myofibroblasts.

**Figure 3:**
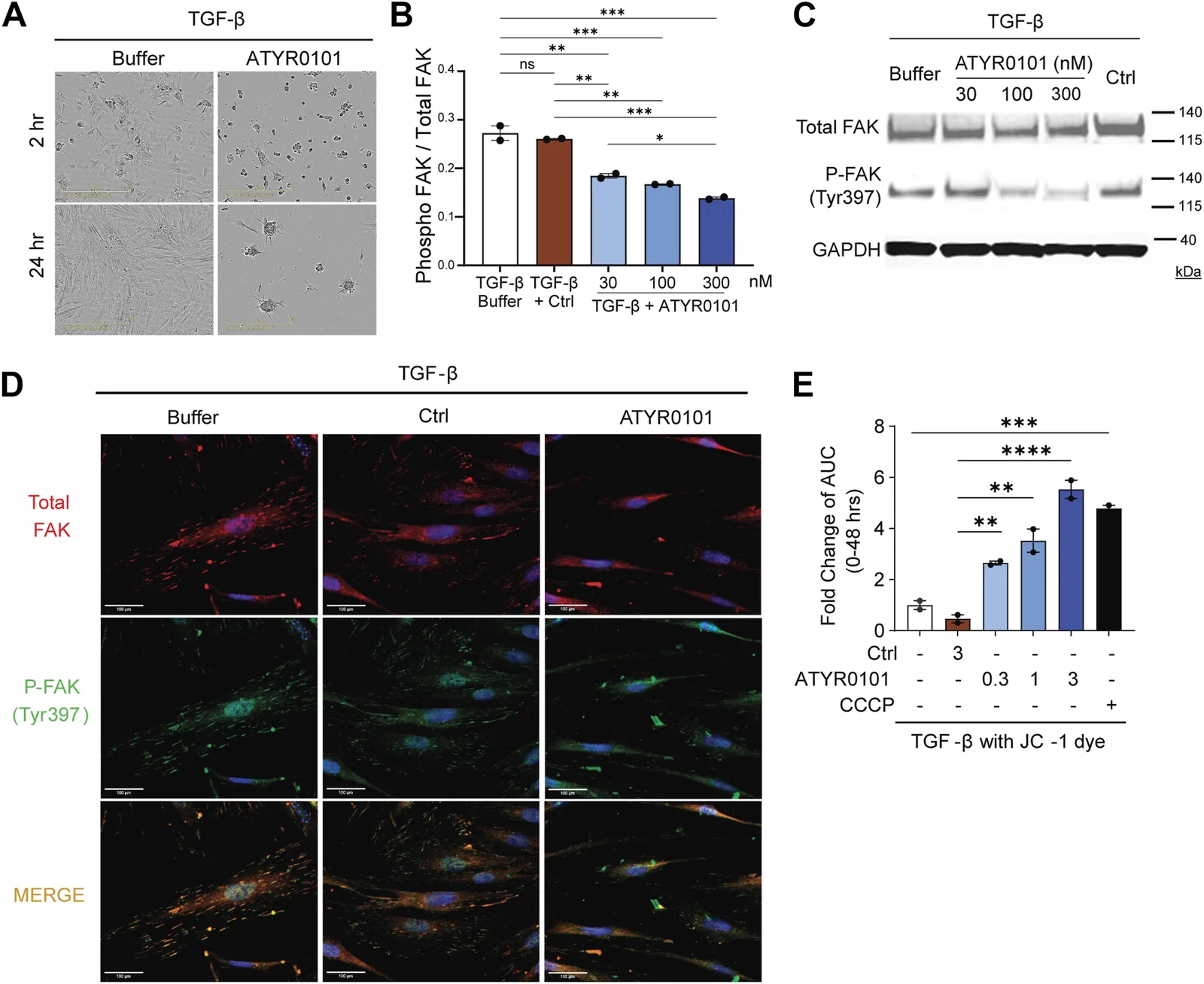
**Impaired FAK activation is found in ATYR0101-treated myofibroblasts.** (*A*) Morphology of ATYR0101 treated WI-38 cells. WI-38 cells treated with TGF-β buffer alone or in the presence of ATYR0101 were lysed 2 hours post-treatment. The activation of FAK was quantified by determining the ratio of phosphorylated (pY397) to total FAK *via* ELISA (*B*) or western blotting (*C*). Data are representative of 3 independent measurements. *D*, cellular localization of focal adhesion kinase in ATYR0101-treated WI-38 myofibroblasts. WI-38 cells were seeded overnight before treated with ATYR0101 or control protein in TGF-β buffer. Cells were fixed, permeabilized and stained with anti-total FAK (*top*), and anti-phosphorylated (pY397) FAK (*middle*) antibodies. Merged images are shown in the *bottom panel*. The arrow indicates the colocalized signal. Data are representative of 3 independent studies. Scale bars are 100 μm. *E*, mitochondrial membrane potential in ATYR0101 or human IgG4 Fc control protein-treated WI-38 myofibroblasts was detected by JC-1 fluorescent intensity through time-lapse live cell imaging. Quantification was performed as area under curve (AUC) compared to protein control after baseline subtraction. Data are representative of 3 independent measurements. CCCP (10 μM) was used as a positive control.

### ATYR0101-induced myofibroblast apoptosis is dependent on LTBP-1 and active TGF-β signaling

To investigate the functional impact of ATYR0101-LTBP-1 binding, we assessed morphological changes as well as annexin V activity in ATYR0101-induced myofibroblast apoptosis in the presence or absence of recombinant LTBP-1. The myofibroblast apoptotic phenotype and subsequent cell death resulting from ATYR0101 treatment was entirely blocked by co-treatment with recombinant LTBP-1. A suppressed annexin V signal was observed in LTBP-1 and ATYR0101 co-treated WI-38 myofibroblasts over a 7-day time-course study compared to ATYR0101 alone (Fig. 4*A*). These findings suggest that ATYR0101-induced myofibroblast apoptosis is LTBP-1-dependent.

**Figure 4:**
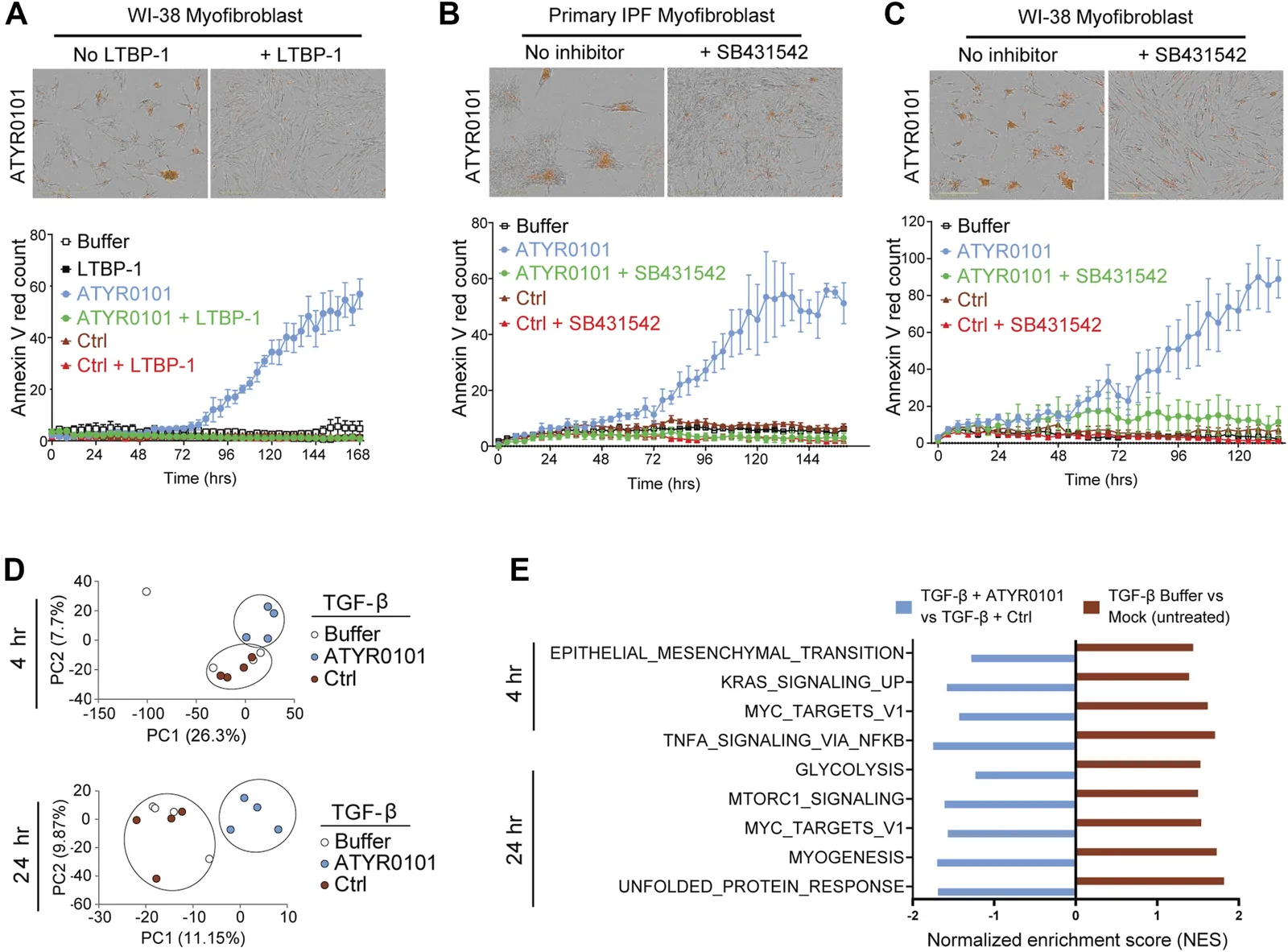
**ATYR0101-induced myofibroblast apoptosis is TGF-β-dependent and relies upon LTBP-1 interaction.***A*, WI-38 fibroblasts were treated with ATYR0101 (1 μM) or a human IgG4 Fc control protein in the presence of TGF-β and then sequestered with recombinant human LTBP-1 (500 nM). Representative data showing morphological changes on day 4 (*left*) and annexin V signal changes (*right*) quantified in real-time on Incucyte. *B* and *C*,TGF-β treated primary IPF fibroblasts (*B*) or WI-38 cells (*C*) were treated with ATYR0101 or human IgG4 Fc control protein (1 μM) in the presence or absence of TGF-β receptor inhibitor SB431542 (3 μM). Annexin V signal changes are measured over a time course of incubation. Scale bars are 400 μm. *D*, principal component analysis of gene expression patterns in WI-38 cells treated with TGF-β buffer alone (*white*), ATYR0101 (*blue*) or human IgG4 Fc control protein (*red*) at 1 μM in the presence of TGF-β for 4 h or 24 h. *Circles* represent 4 biological replicates for each treatment. *E*, normalized enrichment scores (NES) of 50 hallmark pathways by GSEA that compares the transcriptome of ATYR0101-treated TGF-β-differentiated WI-38 myofibroblasts *versus* TGF-β or control protein-treated myofibroblasts (*blue* bars) or TGF-β-differentiated *versus* untreated WI-38 cells (*red bars*). Significantly upregulated pathways are shown in positive NES, and significantly downregulated pathways are shown in negative NES. *p* < 0.05 and FDR q-value < 0.2. n = 4 biological replicates for each treatment. Data are presented as mean ± SEM. n = 3 independent studies. ∗*p* < 0.05, ∗∗*p* < 0.01, and ∗∗∗*p* < 0.001 by one-way ANOVA followed by Tukey’s multiple group comparisons test.

To determine whether the inhibition of TGF-β signaling affects ATYR0101-induced myofibroblast apoptosis, we treated primary IPF patient fibroblasts and WI-38 cells with TGF-β receptor inhibitors prior to ATYR0101 treatment in the presence of TGF-β. Two TGF-β receptor inhibitors were used in this study. SB-431542 selectively inhibited TGF-β receptor−activin receptor-like kinase (ALK) 4, 5, and 7, which blocks all TGF-β and activin-induced Smad signaling pathways (28), whereas SB-525334 inhibited ALK5 in TGF-β-induced phosphorylation and nuclear localization of Smad2/3 (29). Interestingly, a complete loss of the ATYR0101 induced myofibroblast apoptotic phenotype and impeded annexin V signal were found in primary IPF patient myofibroblasts (Fig. 4*B*) as well as in WI-38 myofibroblasts (Fig. 4*C*) when treated with SB-431542 (Fig. 4, *B* and *C*) or SB-525334 (Fig. S6). These results demonstrate that ATYR0101-induced myofibroblast apoptosis is dependent upon TGF-β signaling. Moreover, genome-wide whole-transcriptome analysis (RNA sequencing) showed that ATYR0101-treated WI-38 myofibroblasts had distinct transcriptional profiles compared to control protein− or TGF-β buffer− treated cells (Fig. 4*D*). Unbiased gene set enrichment analysis (GSEA) (30, 31) of 50 hallmark pathways demonstrated that ATYR0101 significantly down-regulated multiple TGF-β-activated pathways, including epithelial-mesenchymal-transition, glycolysis, MYC and mTOR signaling (*p* < 0.05 and false discovery rate (FDR) q value < 0.2) (Fig. 4*E*). These pathways are critically involved in fibrosis development, suggesting ATYR0101 as a novel modulator of TGF-β-signaling in fibrosis. Altogether, this unique ATYR0101-induced myofibroblast apoptosis activity was determined to be dependent upon LTBP-1 interaction, TGF-β activation and resulted in downstream gene expression changes.

### ATYR0101 exhibits anti-fibrotic activity in animal models of fibrosis

We next wanted to determine the effects of ATYR0101 on lung fibrosis *in vivo*. Consistent with the *in vitro* expression of LTBP-1 by WI-38 human pulmonary fibroblasts, the expression of LTBP-1 was readily detectable in the parenchyma of healthy mouse lung (Fig. 5, *A* and *C*). Analysis of LTBP-1 expression in lungs of bleomycin-treated mice showed a slight but significant increase in LTBP-1 expression in the fibrotic lung overall, as well as pronounced expression in fibrotic foci (Fig. 5, *B* and *C*). Having confirmed that the target of ATYR0101 was expressed in both normal and fibrotic lungs, we wished to verify that ATYR0101 is both present in circulation and in the lung following dosing. Pharmacokinetic analysis of ATYR0101 showed that ATYR0101 is present in circulation 24 h post-dosing *via* intraperitoneal injection, and that it can also be detected in the bronchoalveolar lavage fluid (BALF) (Fig. S7, *A* and *B*). Duplex immunofluorescence analysis further showed that ATYR0101 colocalizes with LTBP-1 in fibrotic mouse lung tissue (Fig. S7*C*). We next utilized the bleomycin model to examine the pharmacological activity of ATYR0101. Bleomycin-induced mice treated with ATYR0101 showed a significant reduction in both collagen content and Ashcroft score as compared to vehicle control, indicating that ATYR0101 treatment results in a marked decline in pulmonary fibrosis in this model. Nintedanib which was used as a model control in this study was more effective at reducing collagen content, whereas ATYR0101 treatment significantly reduced Ashcroft score—which evaluates both collagen content and the remodeling of the lung tissue (32) (Fig. 5, *E*–*G*). Further, the frequency of α-Smooth Muscle Actin (α-SMA) positive cells is reduced by ATYR0101-treatment as compared to vehicle, suggesting that there are fewer myofibroblasts in the lungs of ATYR0101-treated animals (Fig. 5*D*).

**Figure 5:**
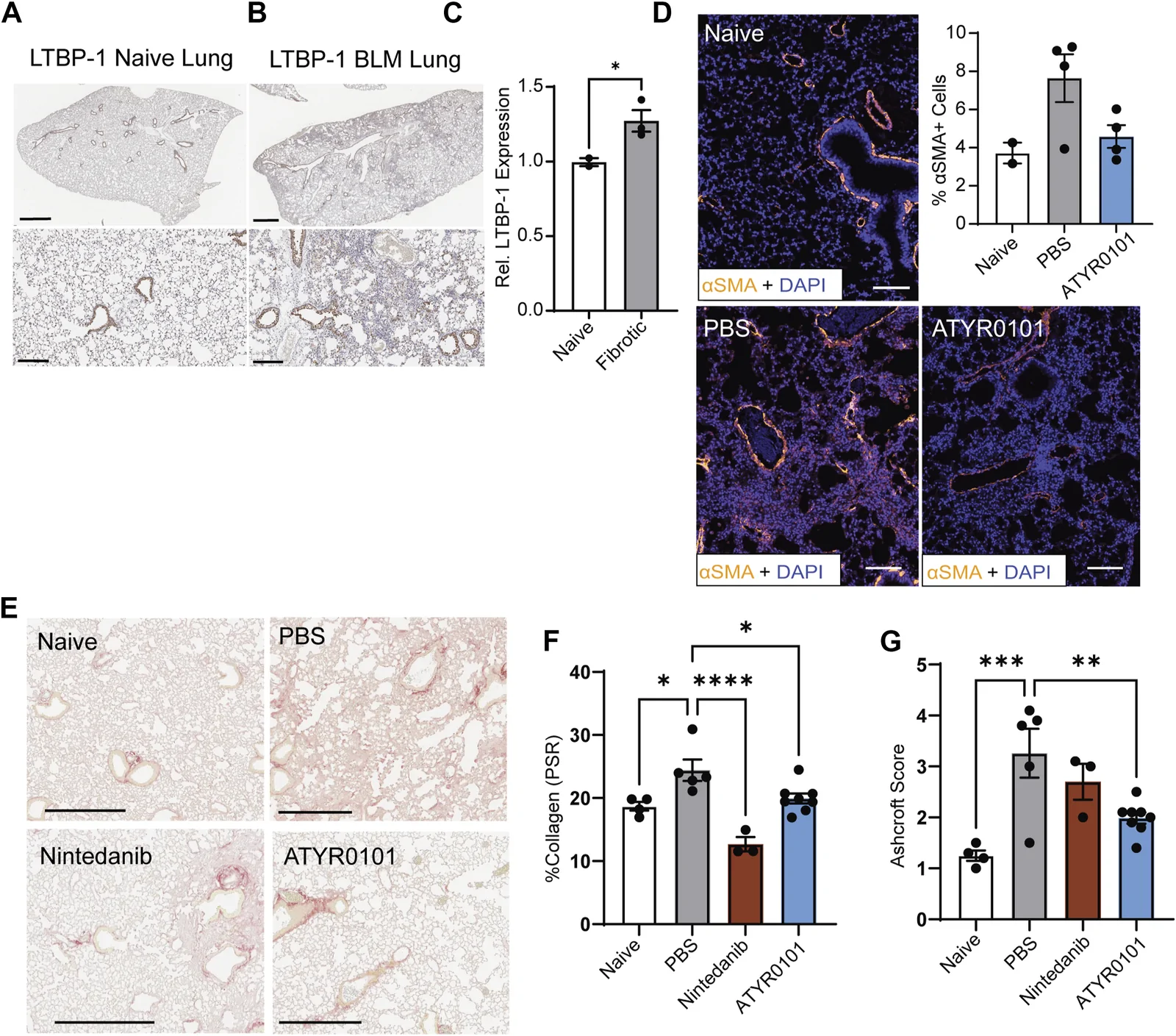
**ATYR0101 reduces fibrosis and the frequency of α-SMA + cells in the bleomycin lung fibrosis model.***A* and *B*, using immunohistochemistry, LTBP-1 expression was detected in the bronchioles and alveoli of normal healthy mouse lungs as well as fibrotic foci and the surrounding parenchyma of bleomycin-induced mouse lungs 21 days post-induction. *C*, quantification *via* HALO showed a significant increase in relative LTBP-1 expression in BLM-treated lungs. Data are shown as mean ± SEM. n = 2 (naïve) n = 3 (fibrotic) biological replicates. ∗*p* < 0.05 by unpaired *t* test with Welch’s correction. Scale bars are 1 mm (*top panels*) or 200 μm (*bottom panels*. *D*, representative immunofluorescence images of α-SMA expression in the naïve lung and fibrotic foci of PBS-treated or ATYR0101-treated bleomycin-induced lungs. Quantitative analysis showed that ATYR0101 reduces the frequency of α-SMA + cells in treated animals. Data are shown as the mean ± SEM. n = 2 (naïve), n = 5 (PBS), n = 5 (ATYR0101) biological replicates per group. Scale bars represent 100 μm. *E*, representative PSR-stained images for each treatment group of the bleomycin lung fibrosis efficacy study are shown. Scale bars represent 500 μm. *F* and *G*, lung collagen content and Ashcroft scores for naïve mice and mice treated with either the PBS negative control, nintedanib positive control or ATYR0101. Data are shown as a mean ± SEM. n = 4 (naïve), n = 5 (PBS), n = 3 (Nintedanib), n = 8 (ATYR0101), biological replicates per group. ∗*p* < 0.05, ∗∗*p* < 0.01, ∗∗∗*p* < 0.001, ∗∗∗*p* < 0.0001 by ordinary one-way ANOVA.

To build upon the results in the lung, we next wished to determine whether the anti-fibrotic effects of ATYR0101 extended into additional fibrotic organs. The Unilateral Ureteral Obstruction (UUO) model of renal fibrosis was utilized to examine the effects of ATYR0101 in the kidney, having observed that LTBP-1 expression is increased in the kidneys of UUO mice as compared to naïve mice (Fig. S11, *A–C*). UUO mice treated with ATYR0101 have reduced kidney weights and cortical collagen content as well as a significant reduction in fibrosis as determined by pathologist evaluation (Fig. S11, *D−G*).

### Spatial transcriptomics reveals ATYR0101 reduces fibrosis by activation of restorative pathways

To further interrogate the mechanism by which ATYR0101 treatment reduces lung fibrosis *in vivo* we analyzed samples from the bleomycin lung fibrosis model (Fig. 5, *E*–*G*) using spatial transcriptomics. Dimensional reduction and clustering identified 18 unique cell populations with a defined gene expression profile and spatial location (Figs. 6*A* and S8). Cell type deconvolution using a single-cell RNA-sequencing reference dataset (33) identified the predominant cell lineage within each population, which was then used to name the corresponding cell populations (Table 1 and Fig. S9*A*). Cell populations enriched in the fibrotic (PBS-treated), nintedanib-treated, and ATYR0101-treated bleomycin lung as well as naïve (Untreated) lung were then defined, resulting in the identification of seven distinct phenotypes (Fig. 6*B* and Table 2), each representing one or more cell populations abundant in specific treatment conditions. Interestingly, though both nintedanib and ATYR0101 treatment reduce fibrosis in the bleomycin lung model (Fig. 5, *E*–*G*) the cell populations associated with each treatment are distinct, suggesting that their mechanisms of action may differ. This is exemplified by the Epithelial_Alveolar.5 cell population, which is abundant in both BLM + PBS and BLM + NIN-treated lungs, indicating similarities between both treatment groups not found in ATYR0101-treated lungs. On the other hand, the four populations composing the MOA-specific phenotype indicate similarities between Untreated and ATYR0101-treated lungs not found in BLM + NIN-treated lungs. In line with this, the spatial distribution of cell populations across the lungs of mice under different treatment conditions revealed that cell populations present in ATYR0101-treated lungs more closely resemble those present in the naïve lung than do those of the nintedanib-treated lungs (Fig. 6*C*). Differences in cell composition of ATYR0101-treated and NIN-treated lungs are translated into distinct transcriptomic profiles between these two conditions. Pathway activity analysis showed that the cell populations abundant in NIN-treated lungs (Fibrotic-NIN and NIN-specific phenotypes) have higher transcriptomic activity within inflammation-associated pathways (including NF-kB, TGF-β, and TNF-α) as compared to cell populations abundant in ATYR0101-treated lungs (MOA-specific phenotype) (Fig. S9*B*). Pathway enrichment analysis showed that cell populations from fibrosis-associated phenotypes (Fibrotic, Fibrotic-NIN, and Fibrotic Naive) are marked by upregulated genes enriched for biological processes related to inflammation and fibrosis (Fig. S9, *C* and *D*). The ATYR0101-specific phenotype is characterized by upregulated genes associated with angiogenesis, especially VEGF signaling (Fig. S10). While these pathways have been associated with fibrosis, they are also associated with regeneration (34). Given that the cell populations abundant in ATYR0101-treated lungs bear a close resemblance to naive lung, the activation of VEGF signaling may be associated with the regeneration of injured lung tissue. Taken together, these results suggest that targeting LTBP-1 by ATYR0101 may promote the restoration of the lung to a healthy state.

**Figure 6:**
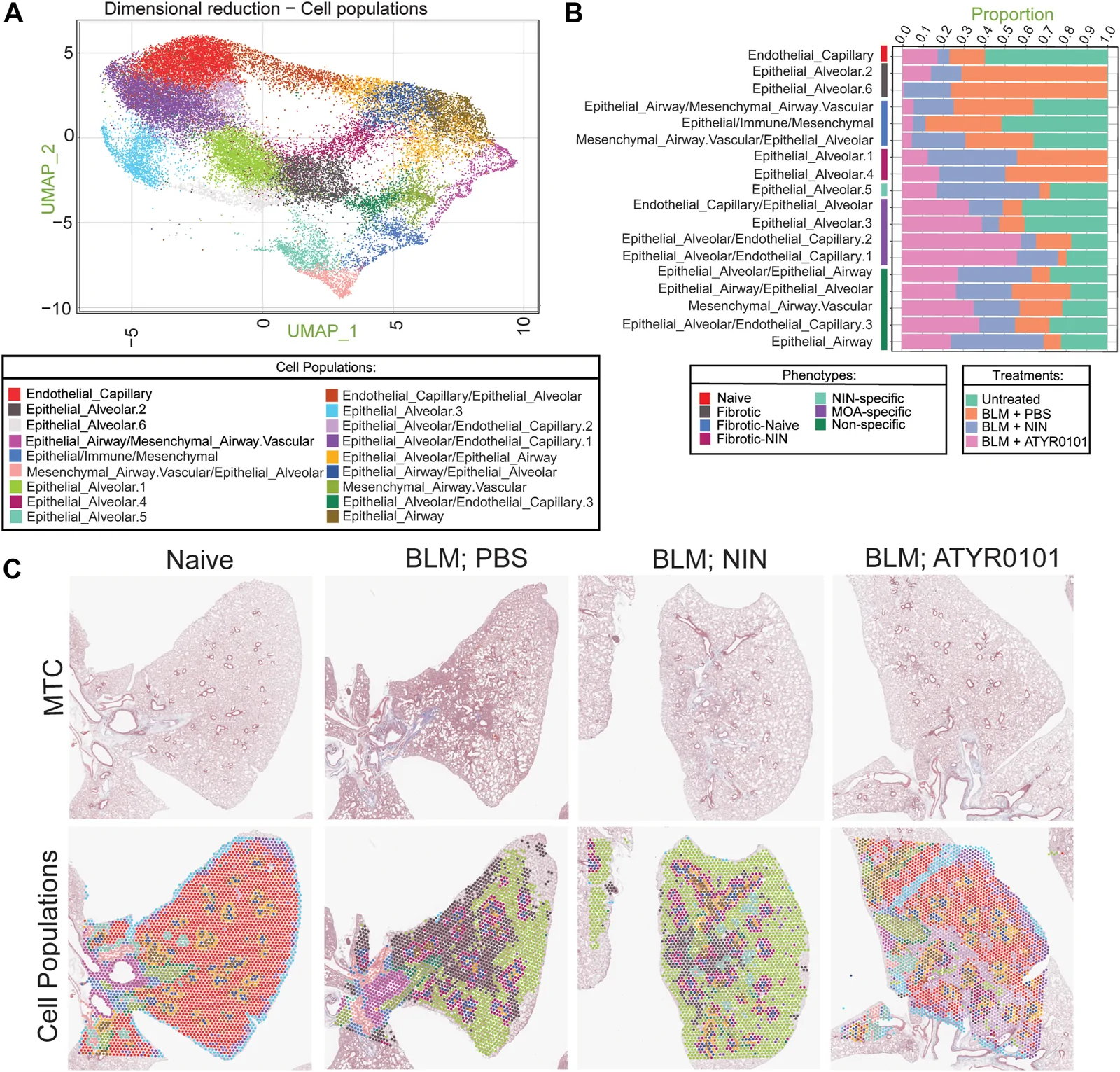
**ATYR0101 promotes a healthy lung phenotype in bleomycin model *via* a mechanism of action that is distinct from nintedanib.***A*, U-map plot of dimensional reduction and clustering identifying 18 unique cell populations. *B*, bar graph illustrating the proportion of spatial transcriptomics (ST) spots from each treatment group within each cell population and their associated phenotype. *C*, representative Masson’s trichrome staining (*top*) and spatial dimensional plots (*bottom*) showing the spatial distribution of the cell populations across all treatment groups.

**Table 1:** Reference scRNAseq lineages and cell types

Lineage	Cell type (abbreviation)	Cell type (full name)
Endothelial_Capillary	CAP1/EPC	CAP1/Endothelial progenitor cell
Endothelial_Capillary	CAP2	Capillary 2 cell
Endothelial_Main	VEC	Venous endothelial cell
Endothelial_Main	AEC	Alveolar endothelial cell
Endothelial_Main	LEC	Lung endothelial cell
Epithelial_Airway	Basal	Basal epithelial cell
Epithelial_Airway	Ciliated/Deuterosomal	Ciliated and Deuterosomal cell
Epithelial_Airway	PNEC	Pulmonary neuroendocrine cell
Epithelial_Airway	Secretory	Airway secretory cell
Epithelial_Alveolar	Epithelial_Alveolar	Epithelial alveolar
Immune_InnateLymphoid	ILC	Innate lymphoid cell
Immune_InnateLymphoid	NK	Natural killer cell
Immune_Lymphoid	B	B cell
Immune_Lymphoid	T	T cell
Immune_Myeloid	AM	Alveolar macrophage
Immune_Myeloid	Basophil	Basophil
Immune_Myeloid	DC	Dendritic cell
Immune_Myeloid	IM	Interstitial macrophage
Immune_Myeloid	Mast	Mast cells
Immune_Myeloid	Megakaryocyte/Platelet	Megakaryocyte and Platelet
Immune_Myeloid	Monocyte	Monocyte
Immune_Myeloid	Neutrophil	Neutrophil
Mesenchymal_Airway/Vascular	ASMC	Airway smooth muscle cell
Mesenchymal_Airway/Vascular	VSMC	Vascular smooth muscle cell
Mesenchymal_Airway/Vascular	Chondrocyte	Chondrocyte
Mesenchymal_Alveolar	AF1	Alveolar fibroblast 1
Mesenchymal_Alveolar	AF2	Alveolar fibroblast 2
Mesenchymal_Alveolar	Pericyte	Pericyte
Mesenchymal_Alveolar	PMP	Proliferative mesenchymal progenitor
Mesenchymal_Alveolar	SCMF	Secondary crest myofibroblast
Mesenchymal_Pleura	Mesothelial	Mesothelial

**Table 2:** Treatment phenotypes and associated cell populations identified by spatial transcriptomics

Phenotype	Treatment where abundant	Treatment where scarce	Cell populations
Naïve	Naïve	BLM + PBS BLM + ATYR0101 BLM + NIN	Endothelial_Capillary
Fibrotic	BLM + PBS	Naïve BLM + ATYR0101 BLM + NIN	Epithelial_Alveolar.2 Epithelial_Alveolar.6
Fibrotic_Naive	Naïve BLM + PBS	BLM + ATYR0101 BLM + NIN	Epithelial_Airway/Mesenchymal_Airway/Vascular Epithelial_Airway/Mesenchymal_Airway/Vascular Mesenchymal_Airway.Vascular/Epithelial_Alveolar
Fibrotic_NIN	BLM + PBS BLM + NIN	Naïve BLM + ATYR0101	Epithelial_Alveolar.1 Epithelial_Alveolar.4
NIN-specific	Naïve BLM + NIN	BLM + PBS BLM + ATYR0101	Epithelial_Alveolar.5
MOA-specific	Naïve BLM + ATYR0101	BLM + PBS BLM + NIN	Endothelial_Capillary/Epithelial_Alveloar Epithelial_Alveolar.3 Epithelial_Alveolar/Endothelial_Capillary.2 Epithelial_Alveolar/Endothelial_Capillary.1
Non-specific	Naïve BLM + NIN BLM + ATYR0101 BLM + PBS		Epithelial_Alveolar/Epithelial_Airway Epithelial_Airway/Epithelial_Alveolar Mesenchymal_Airway.Vascular Epithelial_Alveolar/Endothelial_Capillary.3 Epithelial_Airway

## Discussion

Existing anti-fibrotic therapies, such as nintedanib and pirfenidone, primarily slow disease progression by inhibiting TGF-β signaling and slowing ECM deposition. However, they lack the capacity to reverse established fibrosis (35, 36, 37), potentially because they do not address the persistence of activated myofibroblasts. Our discovery of the DARS-N1/LTBP-1 axis introduces a mechanistically distinct paradigm: the selective induction of myofibroblast apoptosis to promote homeostatic regulation and disrupt the fibrotic cycle. Unlike canonical apoptotic inhibitors (*e.g.*, BCL-XL mimetics) that often exhibit broad cytotoxicity (38, 39, 40), ATYR0101 exploits a specific vulnerability in the myofibroblast-matrix interaction. By targeting LTBP-1 within the ECM, ATYR0101 appears to disrupt anchorage-dependent survival, effectively clearing the cells responsible for the self-perpetuating fibrotic cycle while sparing healthy epithelial cells and undifferentiated fibroblast populations.

Consistent with our *in vitro* findings, our studies also show that ATYR0101 treatment disrupts the cycle of fibrosis *in vivo* across models of fibrosis, and in the bleomycin lung fibrosis model, the presence of α-SMA+ myofibroblasts within the remaining fibrotic foci of ATYR0101-treated lung is reduced. Our spatial transcriptomics analysis reveals that ATYR0101-mediated reduction in fibrosis is driven by a unique molecular program that facilitates a transition toward a restorative cellular environment. While traditional anti-fibrotics often achieve a reduction in extracellular matrix (ECM) deposition without fundamentally altering the underlying fibrotic cellular landscape, ATYR0101 treatment yields a transcriptomic profile that closely approximates the naïve lung. This “restorative” phenotype suggests that ATYR0101 does not merely arrest disease progression but may facilitate a reversal of fibrotic pathology toward homeostatic architecture. We attribute this regenerative effect to a dual-action mechanism characterized by the targeted clearance of myofibroblasts and the concomitant enrichment of VEGF-associated angiogenic pathways. In contrast to multi-kinase inhibitors that inherently suppress vascular remodeling *via* VEGFR blockade, ATYR0101 promotes an environment conducive to healthy revascularization. Our findings suggest that maintaining pro-angiogenic signaling in the context of myofibroblast depletion may be a fundamental prerequisite for returning the fibrotic lung to a functional state. Furthermore, the enrichment of IL-2 signaling in ATYR0101-treated tissues suggests an auxiliary role in promoting immune-mediated homeostasis, potentially *via* activation of regulatory T-cells (Tregs) which have been shown to be anti-fibrotic in the later stages of the bleomycin model (41, 42). This indicates that ATYR0101 does not just halt the TGF-β-driven programs of EMT and glycolysis but proactively reshapes the immune and vascular microenvironment. Beyond that, ATYR0101 markedly down-regulated several TGF-β–driven pathways, notably epithelial–mesenchymal transition (EMT), glycolysis, MYC signaling, and mTOR signaling. Taken together, these findings suggest that angiogenesis may play a crucial role in restoring a fibrotic lung to a healthy state, and that beyond its anti-fibrotic effects, ATYR0101 may also facilitate restoration of normal lung architecture and function.

Our study has several limitations. While we establish LTBP-1 as the requisite extracellular anchor for ATYR0101-mediated myofibroblast apoptosis, the precise molecular composition of the resulting mechanosensory signaling complex remains to be fully elucidated. The interaction between LTBP-1, integrins, and the cytoskeleton is inherently dynamic (43), thus determining how ATYR0101 binding specifically modulates the physical tension required for FAK-mediated survival signaling will require high-resolution structural and biophysical studies. Furthermore, while the reduction in levels of α-SMA + myofibroblasts in the fibrotic foci of lungs treated with ATYR0101 in the bleomycin model is consistent with our *in vitro* mechanistic findings, the rapid kinetics of efferocytosis (clearance of apoptotic cells by resident macrophages) make the direct *in vivo* capture of apoptotic events in the lung challenging. This prevents us from making a temporal “cause-and-effect” link between ATYR0101 binding to LTBP-1 and the subsequent reduction of α-SMA+ myofibroblasts. Future studies utilizing intravital multiphoton microscopy with FRET-based caspase sensors will be necessary to precisely map the temporal kinetics of myofibroblast clearance and the subsequent inhibition of overall fibrosis.

Overall, our data demonstrate that ATYR0101 has significant potential as a novel anti-fibrotic therapeutic candidate with a unique mechanism of action and the possibility to shift treatment of fibrosis from stabilization to repair (Fig. 7). By modulating an evolutionarily conserved physiological pathway, this therapeutic approach could provide a highly selective strategy to eliminate pathogenic myofibroblasts, disrupt the cycle of chronic fibrosis and facilitate the restoration of normal tissue architecture.

**Figure 7:**
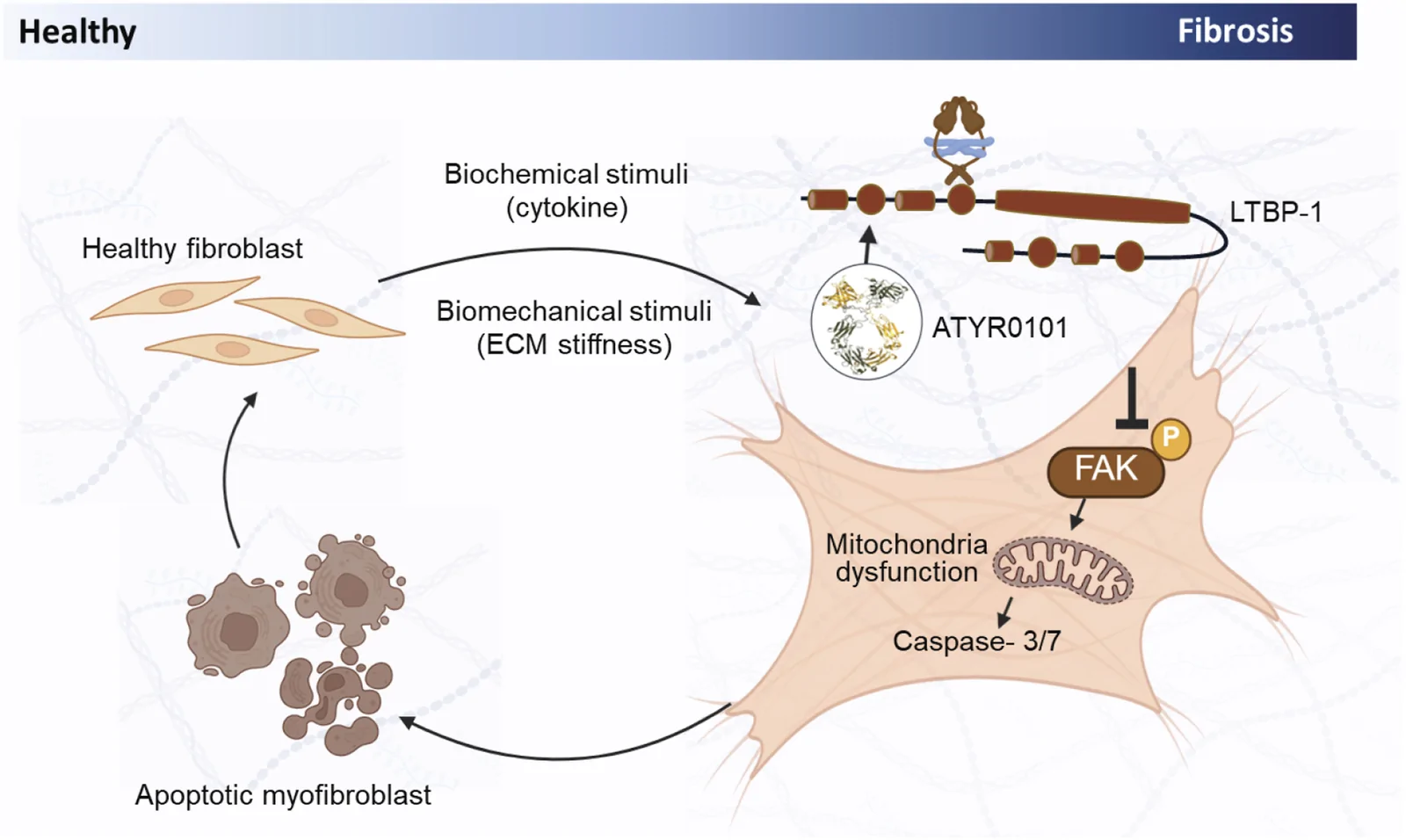
**Mechanism of action for ATYR0101-induced myofibroblast apoptosis.** During tissue damage, fibroblasts differentiate into myofibroblasts in response to growth factor signaling such as TGF-β or biomechanical signals from a stiffened matrix. Activated myofibroblasts are the primary ECM-producing cells during wound healing and fibrosis. Myofibroblasts evade apoptosis in response to pro-survival signals activated by the fibrotic environment that can lead to prolonged fibrosis. ATYR0101 specifically binds to LTBP-1, a large ECM protein that complexes with latent TGF-β and regulates TGF-β bioactivities. The interaction between ATYR0101 and LTBP-1 results in reduced FAK activation, leading to mitochondrial disruption and further elicits caspase-3/7 activation, resulting in myofibroblast apoptosis. Myofibroblast apoptosis induction may contribute to the amelioration of tissue fibrosis and has the potential to restore tissue homeostasis.

## Experimental procedures

### Protein cloning and purification

#### DARS-N1

Aspartate tRNA ligase sequence aa2-154 (accession #P14868.2) was synthesized by IDTdna and cloned into a mammalian expression vector containing a V5 & 6XHis tag. The construct was expressed in Expi293F cells (Gibco, #A14635), harvested, and purified over a nickel excel column equilibrated in 0.05 M Tris, 0.5 M sodium chloride, pH 7.4. The column was washed with 0.05 M Tris, 1M sodium chloride, 0.01 M imidazole, pH 7.4, and the protein was eluted with 0.05 M Tris, 0.5 M sodium chloride, 0.5 M imidazole, pH 7.4. The eluate was concentrated down *via* Amicon Ultracentrifugal filters (Millipore Sigma, #UFC9010) and purified over a Hiload 16/600 Superdex 75 column (Cytiva, #28989333) equilibrated in 1x PBS, pH 7.4. Fractions containing the protein of interest were pooled, concentrated, aliquoted and stored at −80 °C. For the non-binding synthetase fragment control, a fragment from Methionyl-tRNA synthetase (accession #P56192.2) at position 846-900 was cloned, expressed, and purified as described above.

#### ATYR0101

Aspartate tRNA ligase sequence aa3-154 was cloned into a mammalian expression vector containing a rigid proline alanine linker (PA)15 and a human IgG4 tag containing an S228P and L235E mutations to reduce effector function. Cloning was performed using established PCR cloning techniques. Site-directed mutagenesis was performed to mutate R81C, N86C, and C130S in the Aspartate tRNA ligase sequence using standard mutagenesis techniques. The construct was expressed in Expi293F cells (Gibco, #A14635), harvested, and purified over a HiScale 50/20 column packed with MabSelect Sure LX resin (Cytiva, #17547403) equilibrated with 0.02 M sodium phosphate, pH 7.0. The column was washed with 0.02 M sodium phosphate, 0.5 M NaCl, pH 7.0 followed by 0.05 M sodium acetate, pH 5.0. The target protein was eluted with 0.05 M sodium acetate, pH 3.0. The eluate was diluted 1:1 with DI water and neutralized with 7.5% v/v 1M Tris, pH 8.0. The neutralized eluate was filtered through 0.22 uM and purified over an XK 26/20 column packed with Capto S Impact resin (Cytiva, #17371702) equilibrated in 0.02 M sodium phosphate, pH 7.2. The protein was eluted with a 0-100% linear gradient (0.02 M sodium phosphate, 0.5 M NaCl, pH 7.2) over 20 CVs and fractions containing the target protein were combined, filtered through 0.22uM, and processed through a Mustang QXT 5 ml capsule (Cytiva, #XT5MSTGQPM6) equilibrated in 1x PBS, pH 7.4 in flow through mode. The filtrate was concentrated and buffer exchanged into 1x PBS, pH 7.4 with Amicon Ultracentrifugal filters (Millipore Sigma, #UFC9030). The purified protein was filtered through 0.22uM, aliquoted, and stored at −80 °C.

#### LTBP-1 WT

cDNA sequence for human latent transforming growth factor beta binding protein 1 (LTBP1, short isoform), accession #Q14766-2, was ordered from GenScript in pcDNA3.1-C-(k)DYK vector (Genscript, OHu26340D) and a 6XHis tag was added to the plasmid using site-directed mutagenesis (Agilent, #210513). Construct was expressed in Expi293F cells (Gibco, #A14635), harvested, and purified over a nickel excel column equilibrated in 0.05 M Tris, 0.5 M sodium chloride, pH 7.4. The column was washed with 0.05 M Tris, 1M sodium chloride, 0.01 M imidazole, pH 7.4, and the protein was eluted with 0.05 M Tris, 0.5 M sodium chloride, 0.5 M imidazole, pH 7.4. The eluate was concentrated and buffer exchanged into 0.02 M sodium phosphate, pH 7.0 *via* PD-10 desalting columns (Cytiva, #17085101). The sample was purified over a 5 ml HiTrap QHP column (Cytiva, #11001303) equilibrated in 0.02 M sodium phosphate, pH 7.0. The protein was eluted with a 0-100% linear gradient (0.02 M sodium phosphate, 0.5 M NaCl, pH 7.0) over 20 CVs and fractions containing the target protein were combined, concentrated with Amicon Ultracentrifugal filters (Millipore Sigma, #UFC9030), and filtered through 0.22uM. The concentrated eluate was purified over a Hiload 16/600 Superdex 200 column (Cytiva, #28989335) equilibrated in 1x PBS, pH 7.4. Fractions containing the protein of interest were pooled, concentrated, filtered through 0.22uM, aliquoted, and stored at −80 °C.

#### LTBP-1 Fragments

Human LTBP-1 fragments were cloned using standard PCR cloning techniques. Constructs were expressed in Expi293F cells (Gibco, #A14635) and purified by the same method as above. Mouse LTBP-1 (accession #Q8CG19-1) gene sequence was synthesized by Twist Biosciences. Gene fragments were cloned into the same expression vector as LTBP-1-WT plasmid with human LTBP1 signal peptide. Rat LTBP-1 (accession #XP_063118461.1) sequence was synthesized and cloned into pcDNA3.1 vector by Twist Bioscience with the human LTBP-1 signal peptide. Cynomolgus LTBP-1 (accession #XP_045225463.2) sequence was synthesized and cloned into pcDNA3.1 vector by Twist Bioscience with the human LTBP-1 signal peptide. LTBP-1 proteins were expressed in Expi293F cells and purified by the same method as above (LTBP-1 WT).

#### Monoclonal antibodies against DARS-N1 and LTBP-1

Mice were immunized with either recombinant DARS-N1 protein fused to a human serum albumin (HSA) and V5-6xHis tag, LTBP-1(aa1332-1721)-FLAG-6xHis, or LTBP-1(aa1521-1721)-FLAG-6xHis. Antibody-secreting splenocytes were isolated using a fluorescent foci assay. Briefly, NiNTA beads (ThermoFisher, #88831) were coated with antigen of interest, which was either DARS-N1 (V5-6xHis tagged as described above) or LTBP-1(aa347-1721)-FLAG-6xHis, and mixed with AF488 conjugated secondary antibody (ThermoFisher, #A11017) and single cells were picked using a CellCelector automated cell picking system (Sartorius). Antibody variable regions were cloned from the single cells using mouse-IgG specific primer libraries [manuscript in review]. The antibody variable regions were cloned into mammalian expression vectors with mouse IgG1 constant regions. The antibodies were expressed in ExpiCHO cells (Gibco, #A29133), then purified over a HiTrap ProG (Cytiva, #17040501) column followed by a Hiload 16/600 Superdex 200 column (Cytiva, #28989335) equilibrated in 1x PBS, pH 7.4.

### LPS stimulation of macrophages

Human THP-1 cells (ATCC #TIB-202) were differentiated into macrophages with PMA (100 ng/ml, Sigma #P8139) for 48 hrs. Cells were treated with LPS (1 μg/ml, Sigma #L6529) for 8 h and cytosol and media were isolated. Media was concentrated using Amicon Ultra-15 Centrifugal Filter Units (Millipore #UFC901008).

### Liquid chromatography–mass spectrometry analysis

THP-1 conditioned media (100 μg) was separated by SDS-PAGE. Bands spanning 75 kDa to 10 kDa were excised and subjected to standard in-gel tryptic digest procedure. Extracted peptides were acidified and pressure-loaded onto a 100 mm fused silica capillary column containing C18 resin. Peptides were separated with a 160 min organic gradient comprising 10 min of mobile phase A (H_2_0 with 0.1% formic acid), a 110 min gradient to 33.5% mobile phase B (Acetonitrile with 0.1% formic acid), a 10 min gradient to 98% mobile phase B where it was maintained for an additional 10 min and finally finished with a 20 min re-equilibration to mobile phase A. Peptides were eluted into an LTQ-XL ion trap mass spectrometer (Thermo Scientific). A full survey scan was followed by 5 MS/MS scans triggered from a defined parent mass list where the 5 most abundant were selected. This list included m/z: 434.0, 438.5, 487.6, 499.0, 510.1, 511.6, 530.6, 534.6, 567.1, 581.2, 601.7, 604.7, 611.7, 616.2, 640.2, 685.3, 693.3, 700.9, 718.8, 742.1, 755.8, 775.9, 777.9, 832.3, 870.5, 871.9, 878.5, 969.0, 971.1, 977.0, 981.1, 1229.4, and 1256.4. Isolation width and minimal signal requirement were 0.5 and 600, respectively. Spray voltage was set to 1.5 kV and the flow rate through the column was 0.5 μl/min.

### Analysis of mass spectrometry data

RAW files were generated from mass spectra using XCalibur version 2.0 SR2. Ms^2^ spectral data were searched using the SEQUEST algorithm (Bioworks version 3.3.1 SP1) against a custom-made database containing the longest entry from the human IPI database associated with each Ensembl gene identifier resulting in a total of 22,935 unique entries. Additionally, each of these entries was reversed and appended to the database for assessment of false discovery rates. In total, the search database contained 45,870 protein sequence entries (22,935 real sequences and 22,935 decoy sequences). SEQUEST searches allowed for oxidation of methionine residues (16.0 Da), static modification of cysteine residues (57.0Da-due to alkylation), partially tryptic digestion, and a mass tolerance set to ±2 Da for precursor mass and ±1 Da for product ion masses. The resulting ms^2^ spectra matches were assembled and filtered using DTASelect2 (version 2.0.27). For this analysis, tryptic peptides were evaluated using the DTASelect2 software (44). The distribution of Xcorr and deltaCN values for a direct (to the direct database) and decoy (reversed database) were separated by quadratic discriminant analysis. Outlier hits in the two distributions were removed. Spectral matches were retained with Xcorr and deltaCN values that produced a maximum peptide false positive rate of 1% that was derived from the frequency of matches to the decoy reverse database (number of decoy database hits/number of filtered peptides identified × 100). This value is calculated by the DTASelect2 software. In addition, a minimum peptide length of seven amino acid residues was imposed. Such criteria resulted in the elimination of most decoy database hits.

### *In vitro* proteolysis

Purified full length DARS protein (1 μg) was incubated with PMN elastase (50ng-0.8 ng; Innovative Research #HNE) or MMP-12 (50ng-0.8 ng; R&D Biosciences #917-MP) for 30 min at 37 °C. Digested protein was run on SDS-PAGE and stained with Coomassie.

### SDS-PAGE

Purified proteins were resolved on NuPAGE 4-12% Bis-Tris Mini Protein Gels (Invitrogen, #NP0321BOX) along with the SeeBlue Plus2 Pre-stained Protein Standard molecular weight marker (Invitrogen, #LC5926). Samples were kept non-reduced or reduced with 100 mM DTT and heated at 95 °C for 5 min. Gels were stained with InstantBlue Coomassie protein stain (Abcam, #AB1192111000ml).

### Analytical size exclusion chromatography

The overall molecular size, presence of aggregated species, and low molecular weight impurities were determined by analytical SEC HPLC analysis. Purified proteins were loaded on a TSKgel SuperSW3000 4.6 mm × 30 cm column (Tosoh, #0018675) equilibrated in 200 mM Sodium Phosphate, 100 mM Sodium Chloride, 10% Methanol, pH 7.0. 35 μg of purified protein was injected at a flow rate of 0.3 ml/min with a 30 min run time on an Agilent HPLC system.

### Cell culture of lung fibroblasts

WI-38 cells, a human lung fibroblast, were purchased from ATCC (ATCC, #CCL-75). Cells were cultured in EMEM (ATCC, #30-2003) containing 10% fetal bovine serum (FBS) (Gibco, #10082147) and antibiotics (penicillin-streptomycin; Gibco, #15140-122). Primary diseased human lung fibroblasts isolated from an 83-year-old male IPF patient (Lonza, #CC-7231, Batch #21Tl228112) and primary normal human lung fibroblast (NHLF) isolated from a 57-year-old female lung tissue (Lonza, #CC-2512, Batch #21Tl046624) were purchased from Lonza. Both primary lung fibroblast lines were cultured in fibroblast growth medium (FGM-2) (Lonza, #CC-3132). The cells were cultured in a humidified incubator containing 5% CO_2_ at 37 °C. Cells at passage 4 to 8 were assayed for all experiments. Human recombinant TGF-β1 (R&D system, #7754-BH) and TGF-βR1 inhibitors SB431542 (R&D system, #1614) and SB525334 (R&D system, #31211) were used in this study.

### Immunofluorescent staining

WI-38 cells were harvested by trypsinization and seeded onto the fibronectin (10 μg/cm^2^) pre-coated plate (Greiner SensoPlate. Greiner, #655892) in complete medium (7500 cells per well/96-well plate). The next day, culture media was replaced with assay buffer (EMEM with 0.5% FBS, 0.1 mM L-ascorbic acid (MilliporeSigma, #1831-1KG), and antibiotics) supplemented with 10 ng/ml of TGF-β. Cells were cultured for 6 days to establish an ECM. After 6 days, cells were lysed and removed by adding 20 mM ammonium hydroxide to each well for 10 min with gentle plate agitation. The remaining insoluble ECM was then fixed with 4% paraformaldehyde with Hoescht 33,342 (Invitrogen, #H3520) for 15 min at room temperature. The ECM was blocked with 3% BSA in DPBS for 1 h on ice, washed 3x with wash buffer (0.1% BSA), and then stained with 600 nM DARS-N1 or control proteins in staining buffer (1% BSA) for 1 h. The ECM was further stained with anti-V5-AF488 at 2 μg/ml (R&D Systems, #FAB8926G) for protein detection together with anti-LTBP1-APC at 0.4 μg/ml (R&D Systems, #IC388A) and anti-Collagen VI-DyLight 550 at 1:400 dilution (Novus, #NBP2-97126R) for an ECM counterstain. Images were taken at 20x magnification on the InCell Analyzer 2200. Image analysis was generated on FIJI software. For the LTBP-1 knockdown study, WI-38 cells were cultured in the fibronectin pre-coated plates overnight and then replaced in assay buffer containing TGF-β before LTBP-1 siRNA treatment. LTBP-1 siRNA was obtained from Integrated DNA Technologies using the TriFECTa RNAi kit (IDT design ID hs.Ri.LTBP1.13). The siRNA was delivered to cells using Lipofectamine RNAiMAX transfection reagent (Invitrogen, #13778075) following the manufacturers protocol for 96-well plate transfection. The LTBP-1 siRNA and control siRNA treated cells were cultured for 72 hrs to allow the cells to establish an ECM. The ECM staining was performed in the protocol as described above.

For total FAK and phosphorylated FAK staining, WI-38 cells (1.25e4 cells) were seeded in an 8-chamber Millicell EZ Slide (MilliporeSigma cat# PEZGS0816) in serum reduced assay buffer overnight. The next day, media was removed and replaced with ATYR0101 or human IgG4 Fc ctrl supplemented with TGF-β for 2 hrs. After treatment, cells washed, fixed and permeabilized with ice-cold methanol with Hoescht 33,342 (Invitrogen #H3520) for 10 min on ice. Fixed cells were then blocked with 3% BSA in DPBS for 1 h on ice before staining with primary antibodies, rabbit anti-FAK (total) (ProteinTech, 12636-1-AP) and mouse anti-phospho FAK (Y397) (BD, #611723) for 1 h on ice. Cells were further stained with secondary antibodies, Goat anti-Mouse-AF488 (Invitrogen, #A32723) and Goat anti-Rabbit-AF647 (Invitrogen, #A21245) or 1 h on ice before being imaged on the Zeiss Axio Imager M.2.

### Apoptosis assay

After reaching confluence, cells were detached with Accutase (Corning, #25-058-CI) and stained with Caspase-3/7 dye (Sartorius, #4704) or Annexin V dye (Sartorius, #4641) for apoptosis detection. Apoptosis indicating dye-stained cells were seeded at 5000 cells/well in assay buffer (serum reduced buffer with 0.1 mM ascorbic acid) onto 96-well plates (Corning, #3595) for 1-2 hrs before adding treatments. Cells were subsequently treated with ATYR0101 or its equivalent controls (human IgG4 Fc) which were prepared separately in an assay buffer or assay buffer containing 5 ng/ml of TGF-β (TGF-β buffer). Assay buffers containing 0.5% FBS were used in WI-38 and primary NHLF cells whereas 0.1% bovine serum albumin (BSA) was applied to primary IPF fibroblasts. TGF-βR1 inhibitors were treated 30 min before adding TGF-β. Kinetics of cell morphological and apoptosis signals changes were observed and quantified through live cell imaging by Incucyte. Caspase-3/7 activities were also measured using Caspase-Glo 3/7 Assay (Promega, #G8090) at day 2-3 post treatment. For the detection of mitochondria membrane potential, WI-38 cells were treated with ATYR0101 or human IgG4 Fc control proteins in TGF-β buffer for 1 h, then stained with a membrane-permeant JC-1 dye at 2 μM final concentration. Kinetics of mitochondrial depolarization were measured by Incucyte.

For the detection of FAK activation, WI-38 cells were treated with ATYR0101 or human IgG4 Fc control proteins in TGF-β buffer for 2 h, lysed with MSD Tris Lysis Buffer (MSD, #R60TX-3) supplemented freshly with phosphatase and protease inhibitors (MSD, R70AA-1). Cell lysates were analyzed for both total FAK (Invitrogen, #KH00431) and FAK phosphorylated at tyrosine residue 397 (Invitrogen, #KH00441) *via* ELISA. For Western blot, cell extracts were prepared as described above. Whole cell extracts (12 ug determined by Bicinchoninic Acid assay, Pierce, #23227) were analyzed on 10% Bis-Tris SDS-PAGE (Invitrogen, #WBT01026BOX), transferred to PVDF membranes, blocked for 1 h at room temperature followed by overnight incubation with primary antibodies for anti-total FAK (rabbit anti-FAK 1:10,000, Proteintech, #12363-1 AP); anti-Phospho-FAK (rabbit anti-FAK (Tyr397) 1:10,000, Invitrogen, #700255), or GAPDH (Cell Signaling, #2117) at 4 °C. The next day, membranes were incubated with horseradish peroxidase-conjugated secondary antibodies (goat anti-rabbit 1:10,000 from Invitrogen #31460, or goat anti-rabbit 1:3000 from Cell Signaling #7040) before developed with chemiluminescent substrate (Super Signal West Femto: Pierce, #34096).

### AlphaLISA binding assay

For LTBP-1 binding validation, ATYR0101 or a control protein (human IgG4 Fc) was 3-fold serially diluted from 4500 nM to 6.17 nM. 10 μl of serial diluted ATYR0101 or control protein were mixed with 10 μl of rhLTBP-1-FLAG (300 nM) which was prepared in Tris buffer (50 mM Tris-HCl with 0.1% BSA) in a 384-well AlphaPlate (Revvity, #6005359). The protein mixture was incubated on ice for 1 h and protected from light. Subsequently, 10uL of anti-human Fc acceptor beads (Revvity, #AL103) prepared at the concentration of 100 μg/ml in Tris buffer were added to each well. After 1 h incubation at room temperature (RT), 20 uL of anti-FLAG donor beads (50 μg/ml, Revvity, #AS103) were added. The sample mixture was incubated for an additional 1 h at RT in the dark and then analyzed using the AlphaScreen protocol on the PerkinElmer Envision 2103. To validate the binding of LTBP-1 fragments, ATYR0101 or a control protein was 3-fold serially diluted from 1500 nM to 2 nM and then mixed with 300 nM of rhLTBP-1 fragments. Subsequently, the mixture was cultured with anti-human Fc acceptor beads and anti-FLAG donor beads following the procedure described above.

### AlphaLISA competition assay

Biotinylated ATYR0101 protein was diluted in assay buffer, 20 mM HEPES, pH 6.8 with 0.1% recombinant albumin, to a fixed concentration of 0.25 nM and 10uL was added to a 96-well half area AlphaPlate (Revvity, #6002350). Streptavidin Acceptor beads (Revvity, #AL125) were diluted to 20ug/ml and 10uL of acceptor beads were added to the biotinylated AYTR0101. The mixture was covered with a black Top Seal-A (Revvity, #6050173) sealer to block out light and incubated for 1 h at room temperature. Competitors, ATYR0101 and a human IgG4 Fc control protein, and blocking antibodies, anti-DARS, anti-LTBP1 and anti-IgG Fc (Jackson Immuno, #109-007-008) were prepared with a starting concentration of 3000 nM and serially diluted to 0.073 with 4-fold serial dilutions and a final 10-fold dilution. After the serial dilutions, recombinant LTBP1-WT-FLAG was diluted to 5 nM. 10 uL of competitor or blocking antibody were added to the biotinylated ATYR0101-Acceptor bead mixture, followed by 10ul of diluted recombinant LTBP1-WT-FLAG. Plate was resealed and incubated for an additional hour at room temperature. Nickel chelate donor beads (Revvity, #AS101) were diluted to 20ug/ml and 10uL were added, protected from light. The plate was resealed then incubated for 1 h at room temperature. After the final hour, the plate was analyzed using the AlphaScreen protocol on the PerkenElmer Envision 2103.

### RNA sequencing (RNA seq)

WI-38 cells were treated with ATYR0101 or its equivalent controls (human IgG4 Fc) in TGF-β buffer for 4 and 24 hrs as described previously. Cells were lysed on-plate with RLT Buffer containing freshly prepared β-mercaptoethanol followed with total RNA purification using the RNeasy Micro kit (Qiagen, #74004) according to the manufacturer’s manual. RNA concentration and RNA integrity number were measured by TapeStation (Agilent). RNA sequencing and data analysis were performed by Quick Biology (Monrovia, CA) to generate the trimmed mean of the M-values (TMM) normalized counts table. The gene set enrichment analysis (GSEA) was analyzed following the user guide at the GSEA website (30, 31).

### Animal studies

All animal studies were performed in compliance with guidelines approved by the Institutional Animal Care and Use Committee (IACUC) of Explora BioLabs (Animal Care and Use Protocol Number EB17-032-011 and Number EB-17-015-006. Animal housing and experimental procedures were conducted at an AAALAC-accredited Explora BioLabs facility (a Charles River Company). The use of male mice for the bleomycin experiments is due to the development of a robust phenotype with less variability as reported (45). Additionally, in the case of the UUO model, the presence of uterine horns can increase the technical difficulty of identifying and isolating the ureter. The findings are expected to be relevant to both sexes as the models are based on disease states that do not depend on sex-specific hormones or reproductive systems.

### Mouse LPS lung inflammation

Wild type C57BL/6 male mice of 8-10 weeks of age were intranasally administered 50 μg of LPS (Sigma #L6529) on days 1 and 2. On day 3, 24 h post final LPS administration, mice were sacrificed and lungs were flushed twice with 300 μl PBS. Both washes were combined, cells were removed by centrifugation (10 min at 250*g*) and the remaining supernatant (BALF) was stored frozen at −20 °C. BAL cells were counted by a hemacytometer.

### Bleomycin lung fibrosis model

Male C57BL/6 mice were anesthetized to achieve a deep surgical plane and administered a 1.75 U/kg dose of bleomycin in 3 ml/kg saline *via* the oropharyngeal route on day 0. Mice were randomized and placed into treatment groups with 8 mice each. Mice were dosed with 10 mg/kg ATYR0101 *via* the intraperitoneal (IP) route three times per week beginning at day 0. In the positive control arm, mice were dosed with 60 mg/kg Nintedanib *via* daily oral gavage beginning on day 7. In the negative control arm, mice were dosed with 10 ml/kg PBS *via* the IP route three times a week beginning at day 0. Mice were euthanized on day 21, lungs were inflated with cold PBS, removed, and fixed in 10% neutral buffered formalin (NBF) for histological analysis. Formalin fixed tissues were paraffin embedded and sectioned at 4 μm and stained with either picrosirius red or Masson’s trichrome. Quantitative image analysis was performed using HALO to determine the percentage of collagen per area. In parallel, Ashcroft score was determined by an independent veterinary pathologist. To examine ATYR0101 concentrations in serum and BALF in the bleomycin model, fibrosis was induced in the manner described above, and mice were randomized and placed in treatment groups with 5 mice each. Mice were dosed with 30 mg/kg ATYR0101 or 10 ml/kg PBS *via* the IP route three times a week beginning day −1. Mice were euthanized on day 7, blood was collected *via* cardiac stick and processed for serum, and lungs were inflated with 0.8 ml cold PBS and then aspirated for BALF collection.

### Unilateral ureteral obstruction model

Irreversible unilateral ureteral obstruction was performed as described (46). Briefly, male C57BL/6 mice were anesthetized to achieve a deep surgical plane; the surgery site was clipped and then scrubbed with betadine and 70% isopropanol alternating each three times and ending with isopropanol. A 2 cm midline laparotomy incision of the avascular linea alba using tissue separating scissors was made to gain access to the peritoneal cavity. The left ureter was exposed with sterilized cotton-tip applicators and ureteric obstruction was created by ligating the ureter twice with sterile black silk sutures between the bladder and renal pelvis. The peritoneum was then closed and sutured, and the skin was approximated using metallic skin clips. Five mice were used per treatment group. Mice were dosed with either ATYR0101 at 30 mg/kg or PBS at 10 ml/kg *via* the IP route three times a week beginning at day 0. Animals were euthanized on day 10, the left kidneys were removed, weighed and fixed in 10% NBF for histological analysis. Histological analysis and scoring were performed as described above.

### Pharmacokinetics

Wild-type BALB/c females at 8-10 weeks of age were used to evaluate the pharmacokinetic profiles of DARS-N1 and ATYR0101. Animals were administered a single 1 mg/kg dose of either DARS-N1 or ATYR0101 systemically. Blood samples were collected at 1, 4, 24, 48, 96, and 168 h post-dosing (n = 3 mice per time point). Submandibular bleeds were performed for all time points except for the terminal 168-h time point which was performed by cardiac puncture. Blood was collected in serum separator tubes and centrifuged at 5000 rcf for 6 min at 4 °C to isolate serum. Serum samples were flash-frozen on dry ice and stored at −80 °C until analysis.

### Immunohistochemistry and immunofluorescence of tissues

#### LTBP-1 Immunohistochemistry

All stainings were performed at HistoWiz, Inc. (Long Island City) using the Leica Bond RX automated stainer (Leica Microsystems) with a standardized operating procedure and fully automated workflow. Samples were processed, paraffin-embedded, and sectioned at 4 μm. Slides were dewaxed using xylene and alcohol-based solutions. Epitope retrieval was performed *via* heat-induced epitope retrieval (HIER) using a citrate-based pH 6.0 solution (Leica Microsystems, #AR9961) at a 1:1000 dilution for 20 min at 95 °C. Sections were incubated with peroxide block buffer (Leica Microsystems), followed by incubation with rabbit polyclonal anti-LTBP-1 antibody (Abcam, #ab78294) at a 1:1500 dilution. Detection was carried out using the Bond Polymer Refine Detection Kit (Leica Microsystems), including polymer HRP, DAB Refine, and hematoxylin, following the manufacturer’s protocol. Slides were dried, coverslipped using the Tissue-Tek Prisma Coverslipper, and scanned at 40X magnification with the Leica Aperio AT2 slide scanner (Leica Microsystems).

#### α-SMA and cleaved caspase-3 (CC3) duplex immunofluorescence

All staining procedures were performed at HistoWiz, Inc. (Long Island City) using the Leica Bond RX automated stainer (Leica Biosystems) following a standardized operating procedure and fully automated workflow. Samples were paraffin-embedded and sectioned at 4 μm. Slides were dewaxed using xylene and alcohol-based solutions. Epitope retrieval was performed by HIER using a citrate-based pH 6.0 solution (Leica Biosystems, #AR9961) for 20 min at 100 °C. Slides were first incubated with peroxide block buffer (Bond Polymer Refine Detection Kit, Leica Biosystems) for 10 min, followed by 1X Antibody Diluent/Block (Akoya Biosciences) for 20 min. The first target, alpha smooth muscle actin (α-SMA), was stained using a rabbit polyclonal anti-α-SMA antibody (Abcam, #ab5694) at a 1:1000 dilution for 60 min, followed by anti-rabbit HRP-conjugated polymer (Bond Polymer Refine Detection Kit, Leica Biosystems) for 30 min. Signal detection was achieved with Opal 620 tyramide (Akoya Biosciences) at a 1:150 dilution in 1X Plus Automation Amplification Diluent, incubated for 30 min. Antibody complexes were then stripped using Bond ER Solution 2 (Leica Biosystems) at 96 °C for 10 min. Slides were re-blocked with 1X Antibody Diluent/Block (Akoya Biosciences) for 20 min. The second target, cleaved caspase-3 (CC3), was stained using a rabbit polyclonal anti-cleaved caspase-3 (Asp175) antibody (Cell Signaling Technology, #9661) at a 1:300 dilution for 60 min, followed by anti-rabbit HRP polymer for 30 min. Detection was performed with Opal 520 tyramide (Akoya Biosciences) at a 1:150 dilution, incubated for 30 min. A second stripping step was performed using Bond ER Solution 1 (Leica Biosystems) at 96 °C for 10 min. Finally, slides were incubated with 1X Spectral DAPI (Akoya Biosciences) at room temperature for 20 min. After staining, slides were air-dried, coverslipped using the Leica CV5030 with ProLong Diamond Antifade Mountant (Invitrogen, #P36970) and scanned at 20X magnification using the Akoya Vectra Polaris/PhenoImager HT fluorescent slide scanner (Akoya Biosciences).

#### LTBP-1 and Fc duplex immunofluorescence

All staining procedures were performed at iHisto, Inc. using a previously optimized manual staining procedure. Samples were paraffin-embedded and sectioned at 4 μm. Paraffin sections were deparaffinized and rehydrated as follows: 15 min xylene, 15 min xylene, 5 min 100% alcohol, 5 min 100% alcohol, 5 min 70% alcohol. HIER was performed using a citrate-based pH 6.0 solution for 15 min at 110 °C. Once slides were cooled to room temperature, slides were washed 3 times for 5 min each in TBS, TBS, and TBST (Tris-buffered Saline + Tween20), respectively. Sections were treated with a peroxidase blocking solution for 10 min at room temperature. Slides were washed as before. 2.5% normal horse serum was applied to the sections, and sections were incubated for 20 min at room temperature. Primary antibody (LTBP1, Abcam, Cat #ab78294, 1-1500) was diluted in 2.5% normal horse serum was applied to each section and allowed to incubate overnight at 4 °C. The following morning, slides were washed as before. Ready-to-use horse anti-rabbit IgG HRP was applied to each section and allowed to incubate for 1 h at room temperature. Slides were once again washed. TSA-FITC working solution was applied to each section and incubated at room temperature for 10 min. Antibody complexes were then stripped using a citrate-based pH 6.0 solution for 15 min at 110 °C. Sections were blocked with 2.5% Goat Serum for 20 min. The second primary antibody (anti-Human IgG Fcy, Invitrogen, Cat #31142, 1-400) diluted in 2.5% normal goat serum was applied to each section and allowed to incubate overnight at 4 °C. The following morning, slides were washed as before. A conjugated goat anti-rabbit secondary antibody (CY3, 1:500) diluted in TBS was applied, and sections were allowed to incubate for 60 min. Autofluorescence and background were quenched using Sudan Black for 20 min at room temperature. Finally, slides were incubated with a DAPI working solution at room temperature for 10 min. Slides were aqueously cover slipped using Anti-Fade Fluorescence Mounting Medium and scanned at 40x magnification using a Pannoramic Midi II Fluorescent Scanner.

### ELISA for ATYR0101 in serum and BALF

ATYR0101 ELISA analysis was performed by a sandwich immunoassay created in house on the Meso Scale Discoveries (MSD) platform. Mouse serum and BALF samples were diluted to be in the range of the assay standard and reduce matrix interference. The capture antibody utilized was anti-DARS-N1 generated in-house as previously described. The standard curve was prepared by serial dilution of ATYR0101. The detection antibody was a commercially available anti-human Fc conjugated to the sulfo-tag utilized in the MSD platform. Plates were read on the MSD Quick Plex 1300. Data were analyzed in GraphPad Prism 10.1 using a 4 PL curve fit and the interpolated standard curve results.

### Spatial transcriptomics sequencing

Spatial transcriptomics was performed by Alimentiv according to standard operating procedures. Briefly, FFPE blocks for 3 samples per treatment group from the bleomycin lung fibrosis model described previously were used to make two 4 μm serial sections, one stained with H&E and one stained with Masson’s trichrome. H&E and Masson’s trichrome whole slide images were uploaded to Alimentiv’s electronic file repository. All stained tissue sections (either H&E or Masson’s trichrome) were imaged on a 3DHISTECH Pannoramic MIDI II slide scanner. The H&E-stained slides were then moved forward to the 10X Visium CytAssist v2 spatial transcriptomics workflow. During the CytAssist-enabled portion of the workflow, an image of each tissue section was acquired by a CytAssist internal camera while gene expression probes were transferred to spatially barcoded oligonucleotide capture areas on a Visium CytAssist slide. Each CytAssist image was registered to its respective high-resolution H&E image (captured by the MIDI II scanner), providing spatial orientation of the tissue. Following the capture of the gene expression probes by the Visium CytAssist slide, spatial barcodes and unique molecular identifiers (UMIs) were incorporated into the probes by an extension reaction. The spatially barcoded probes were eluted from the Visium CytAssist slide and pre-amplified. The pre-amplified eluate then underwent simultaneous amplification and sample indexing, creating the final NGS library for sequencing the sample. Sequencing was performed on Illumina NovaSeq X plus platform utilizing 25B reagent kits (300 cycle) and NovaSeq X Plus Control Software version 1.2.2. Read configuration was: read 1:150 bp, read 2:10 bp, read 3:10 bp, read 4:150 bp, and each sequencing lane incorporated 1% PhiX sequencing control. Sequencing depth was performed at 100% coverage of spots for 124.8 million total reads per RNA library.

### Spatial transcriptomics data analysis

With aid of space ranger software (Version 4.0.0, 10X genomics), the space ranger count pipeline performed alignment, tissue and fiducial detection, and barcode UMI counting using microscope slides images, sequencing (FASTQ files), and manual alignment files as input. Manual alignment was performed between the Cytassist and the Masson's trichrome images with help from the Loupe Browser software (Version 8.0, 10X genomics), to allow the spatial gene expression data to be properly overlayed onto the adjacent (Masson's trichrome) section. Using R statistical programming language, outputs from space ranger count pipeline (gene expression matrices and 10x Visium images) were used to create Seurat objects with aid of Seurat package (Version 5.0.2) (47). Spatial transcriptomics spots of low quality based on the following metrics: those with 0 UMI or having either a higher percentage of mitochondrial genes or hemoglobin genes as well as a low number of detected genes per cell were filtered out of the dataset with help of the scuttle R package (48). Individual Seurat objects (one per sample) were pre-processed by a standard pre-processing workflow, aiming to normalize the data, finding variable features, scaling the data, and performing dimensional reduction by applying the following Seurat package functions: *NormalizeData*), *FindVariableFeatures*), *ScaleData*), and *RunPCA*). Afterwards, the Seurat objects were integrated with Harmony method (49). Clustering of integrated Seurat object was performed by standard Seurat package functions *FindNeighbors*) and *FindClusters*), and non-linear dimensional reduction was performed with aid of *RunUMAP*) function. The final product of dimensional reduction and clustering was the formation of cell populations, or Seurat clusters with a distinct transcriptomic profile and spatial location. The cellular identity of spatial transcriptomics spots was estimated with the help of the R package Robust Cell Type Decomposition (RCTD) (50) and a reference single-cell RNA-sequencing (scRNAseq) dataset containing annotated cell lineages and cell types from mouse lungs at different developmental stages (Table 1) (33). Only samples from post-natal (n = 14) mice in the scRNAseq dataset were used for cell type deconvolution. Cell populations were subsequently annotated according to the most predominant lineages (up to 70% composition) represented among the deconvoluted spots within each population. The annotated cell populations were further categorized into phenotypes reflecting the association of cell populations with treatment conditions: Untreated, BLM + PBS, BLM + NIN, BLM + ATYR0101. Cell populations were classified into phenotypes according to the predominant treatment(s) represented among their spots: those in which a single treatment accounted for ≥ 60% of spots, or multiple treatments collectively accounted for ≥ 70%. The classification of cell populations into phenotypes is summarized in Table 2. Differentially Expressed Genes (DEGs) expressed by at least 25% of cells within each cell population, having an adjusted *p* value < 0.05, and with a log2 fold change of at least 0.25, were identified with aid of Seurat package function *FindAllMarkers*). Afterward, DEGs were defined as upregulated (log2 fold change > 0.25) and downregulated (log2 fold change < −0.25). Pathway activity analysis was performed to compare the gene expression data from the cell populations with gene sets from the following pathways: Androgen, EGFR, Estrogen, Hypoxia, JAK-STAT, MAPK, NFkB, p53, PI3K, TGF-β, TNF-α, Trail, VEGF, and WNT, with help from the R package Progeny (51). Each cell population was scored for the pathways, allowing for comparisons of pathway activity between them. Pathway enrichment analysis (PEA) was performed using the set of upregulated DEGs as input to perform an Over Representation Analysis (ORA) with the one-sided version of Fisher’s exact test. Accordingly, the list of upregulated DEGs for each cell population was contrasted with the ∼7600 gene sets present in the Human Molecular Signatures Database (MSigDB) Gene Ontology (GO) Biological Process with help from the ClusterProfiler R package (52). The ClusterProfiler function *compareCluster*) was used to identify GO Biological Process specific and common to the different cell populations.

## Data availability

All raw data will be available upon request from the corresponding authors.

## Supporting information

This article contains supporting information.

## Conflict of interest

ATYR0101 is protected by issued and pending US and international patents including; U.S. Patent No. 9896680 entitled Compositions And Methods Comprising Aspartyl-Trna Synthetases Having Non-Canonical Biological Activities; U.S. Patent No. 9816084 entitled Improved Aspartyl Trna Synthetases and PCT Patent Application No. PCT/US2025/028963 entitled Modified Aspartyl-Trna Synthetases.

Authors who are current or past employees of aTyr Pharma may have related financial compensation.

## Figures and Tables

**Figure S1:**
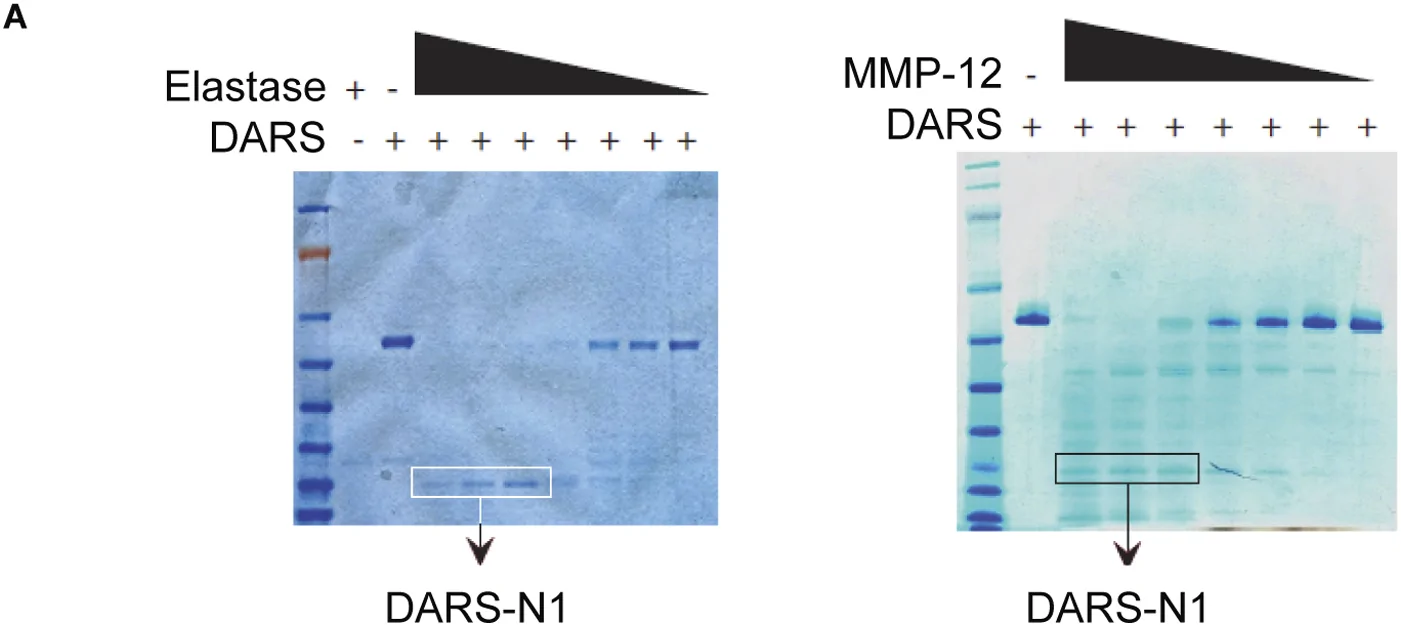


**Figure S2:**
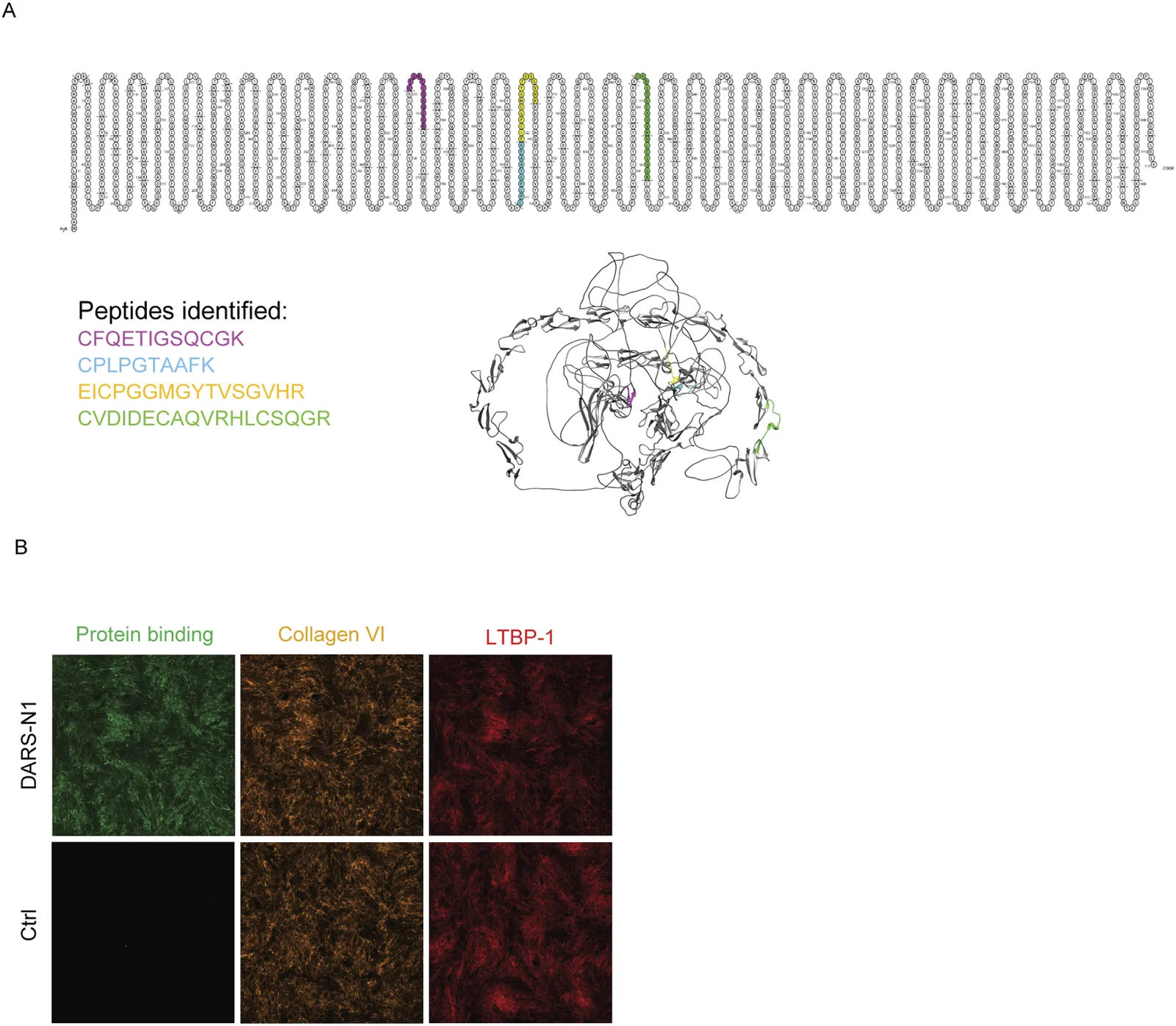


**Figure S3:**
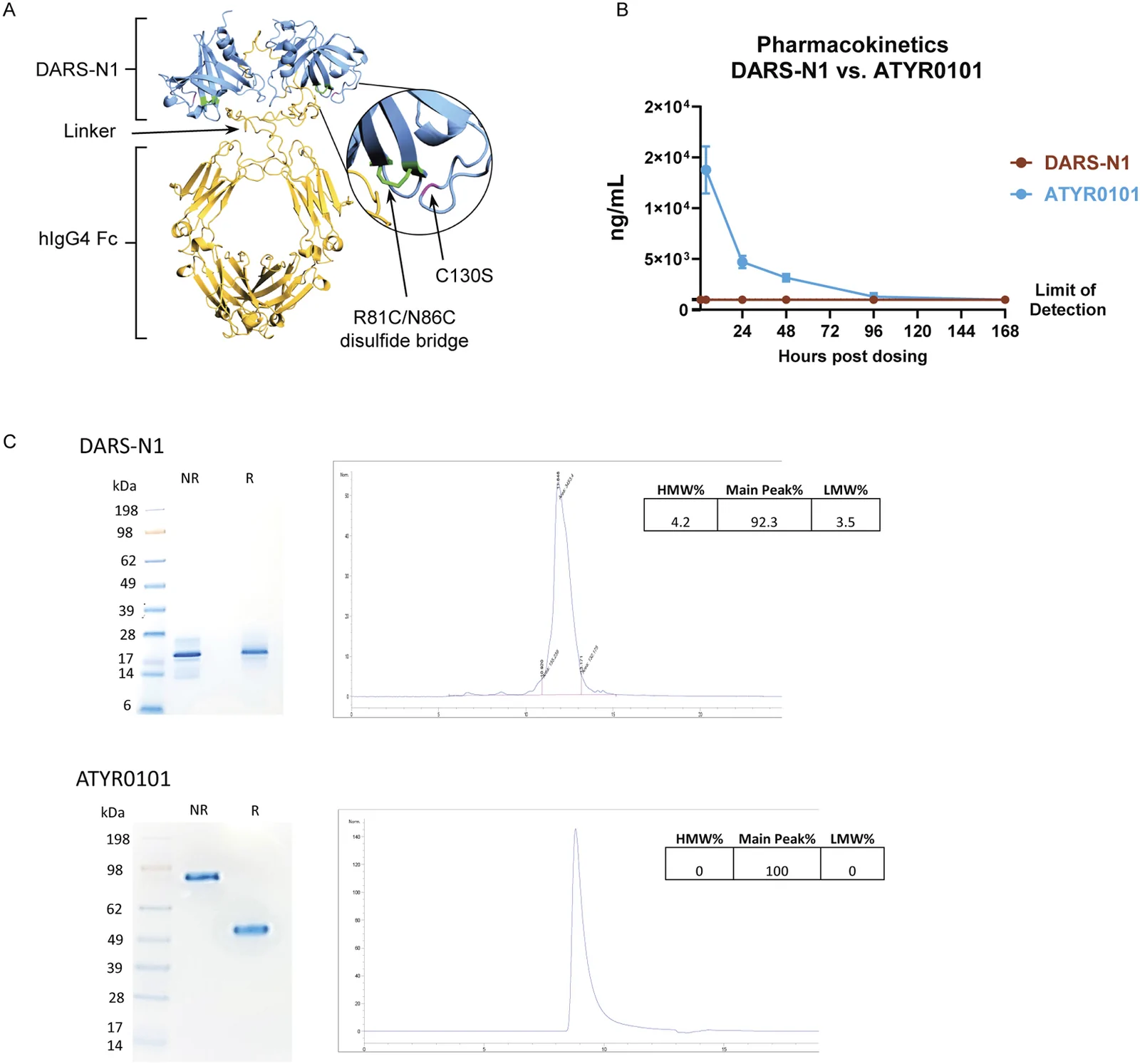


**Figure S4:**
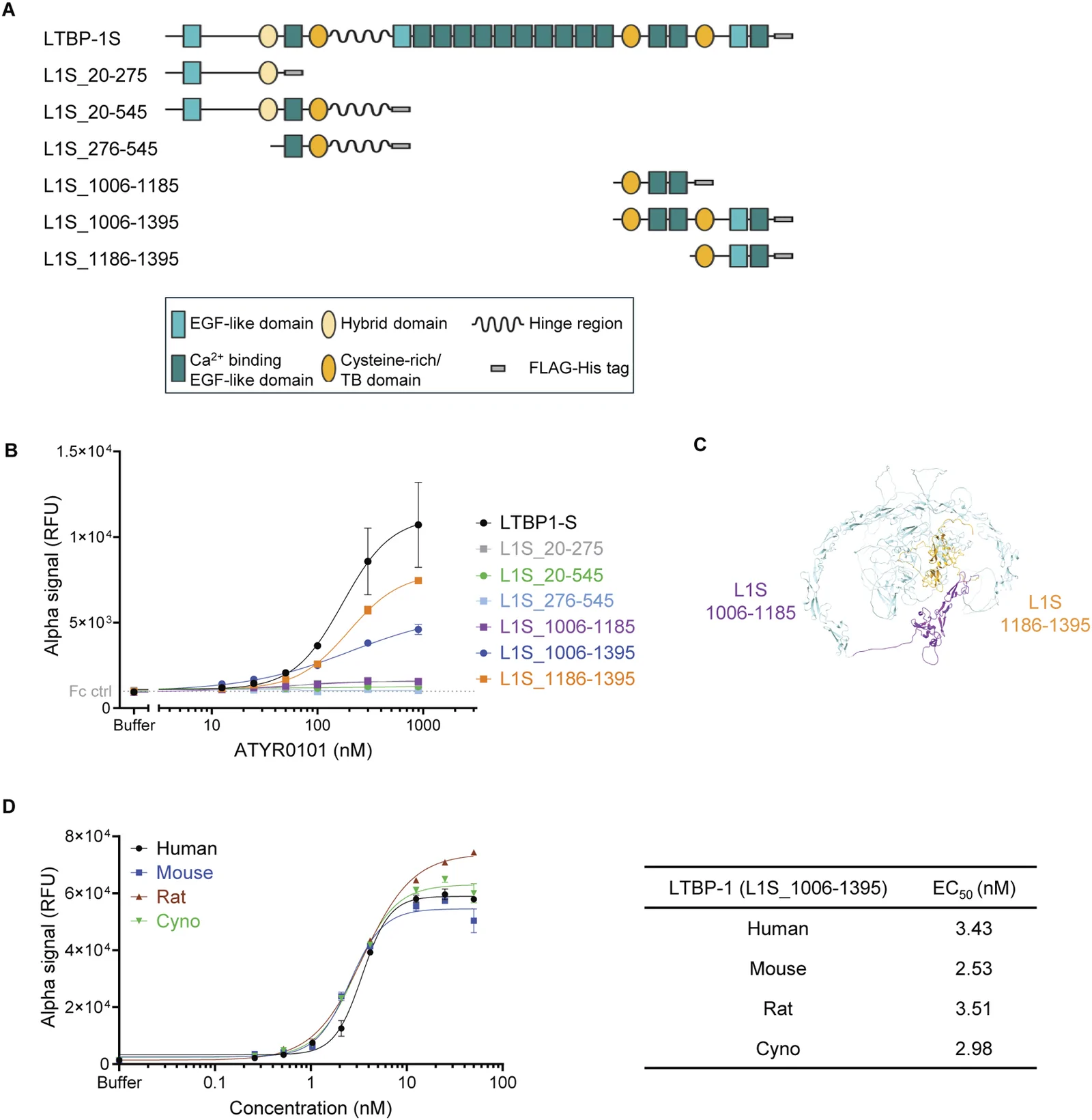


**Figure S5:**
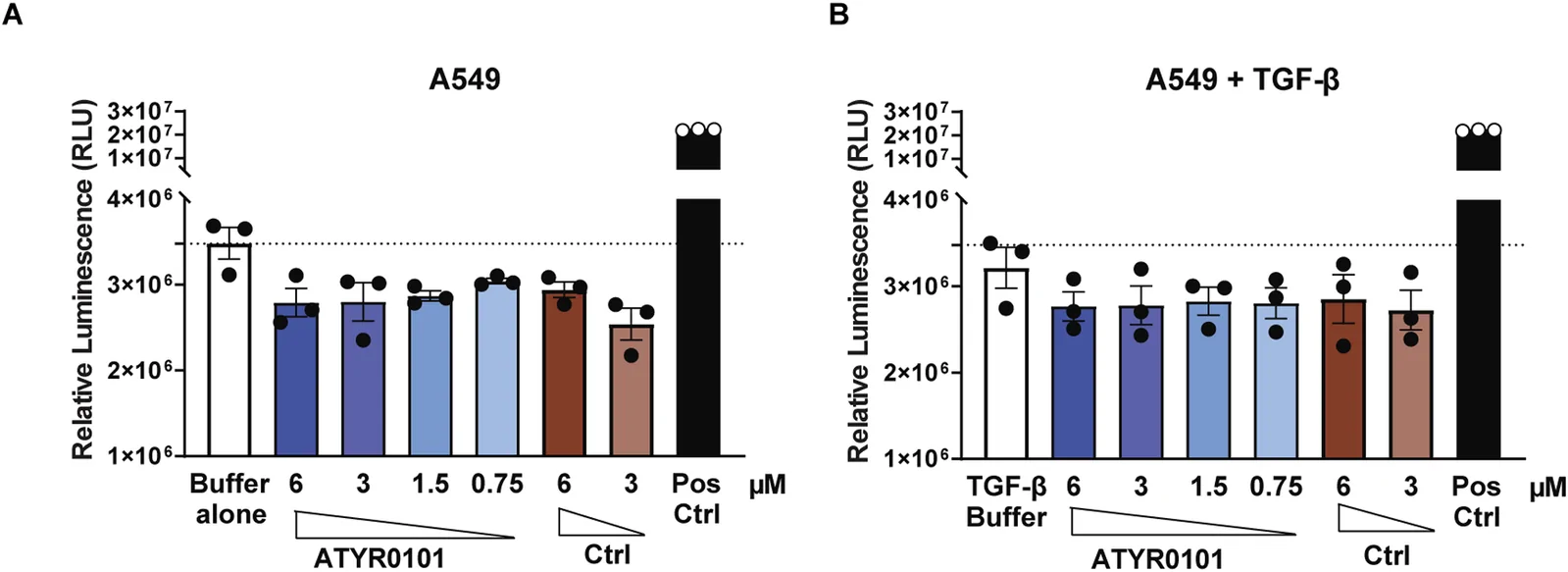


**Figure S6:**
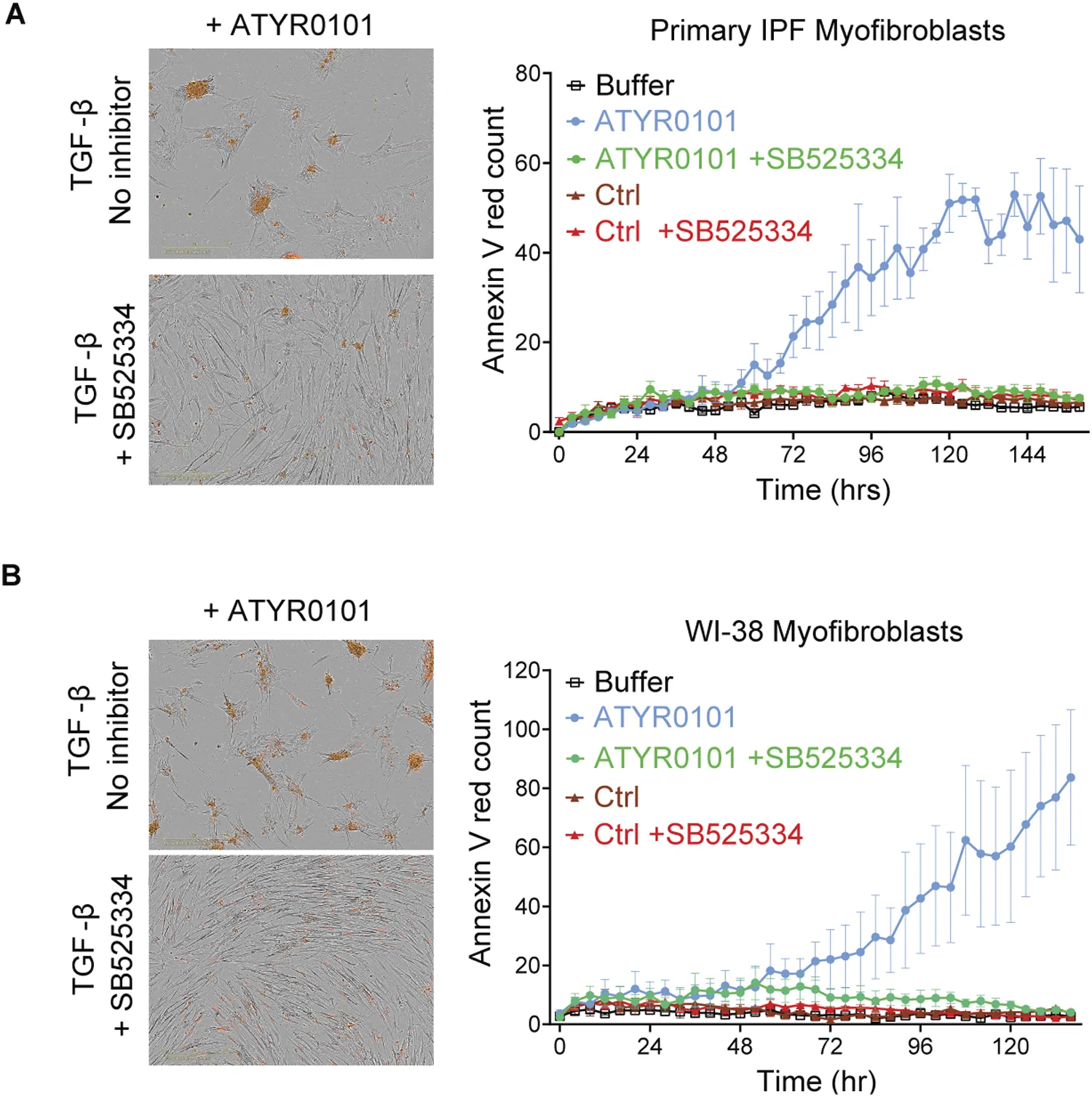


**Figure S7:**
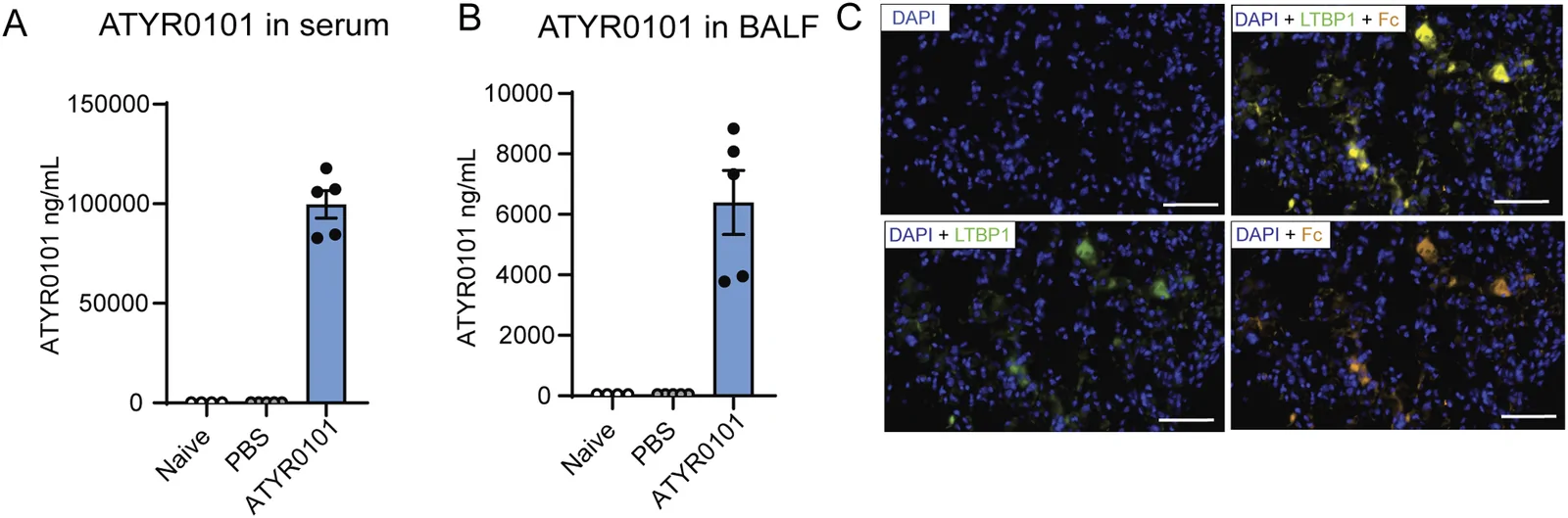


**Figure S8:**
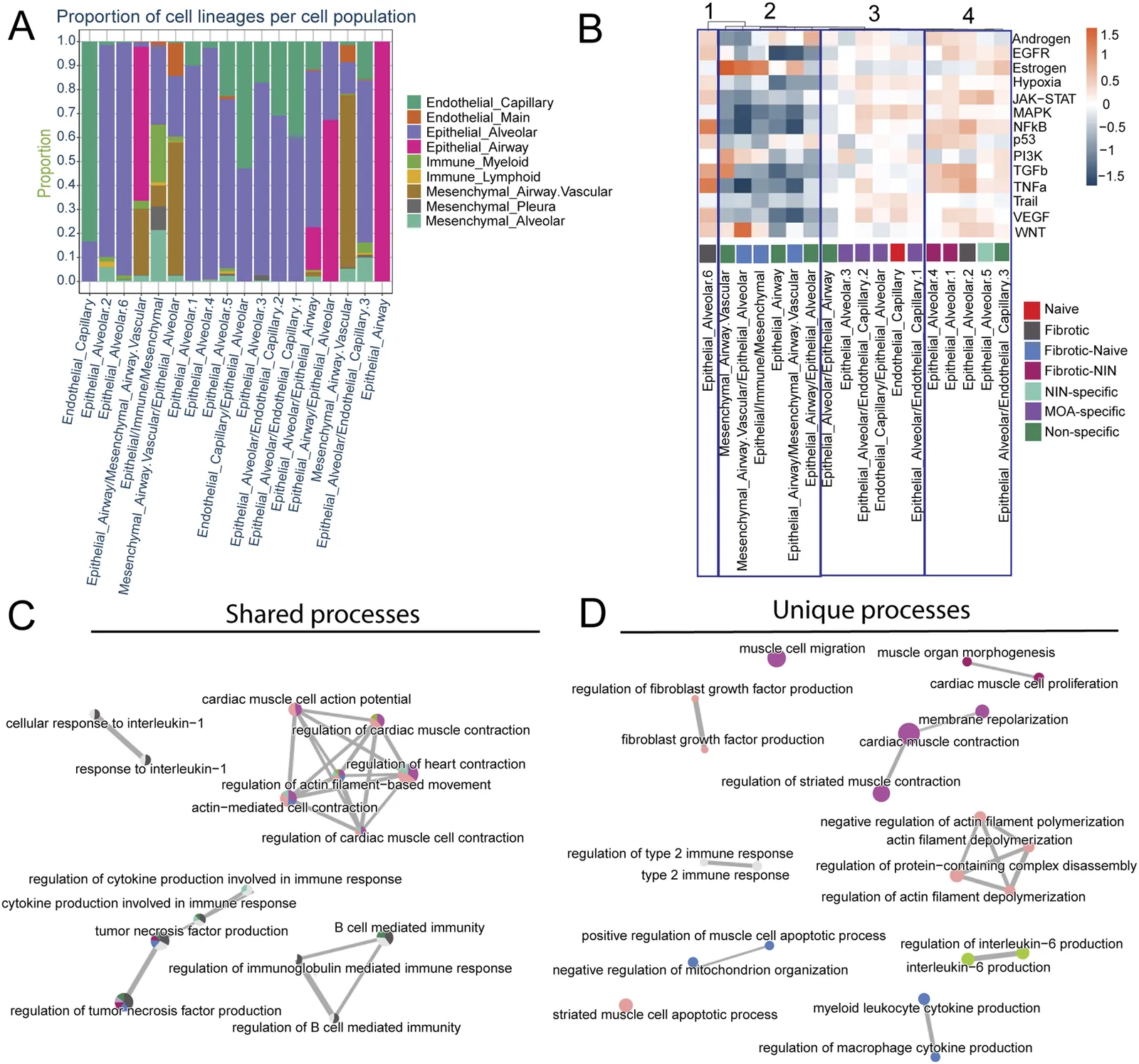


**Figure S9:**
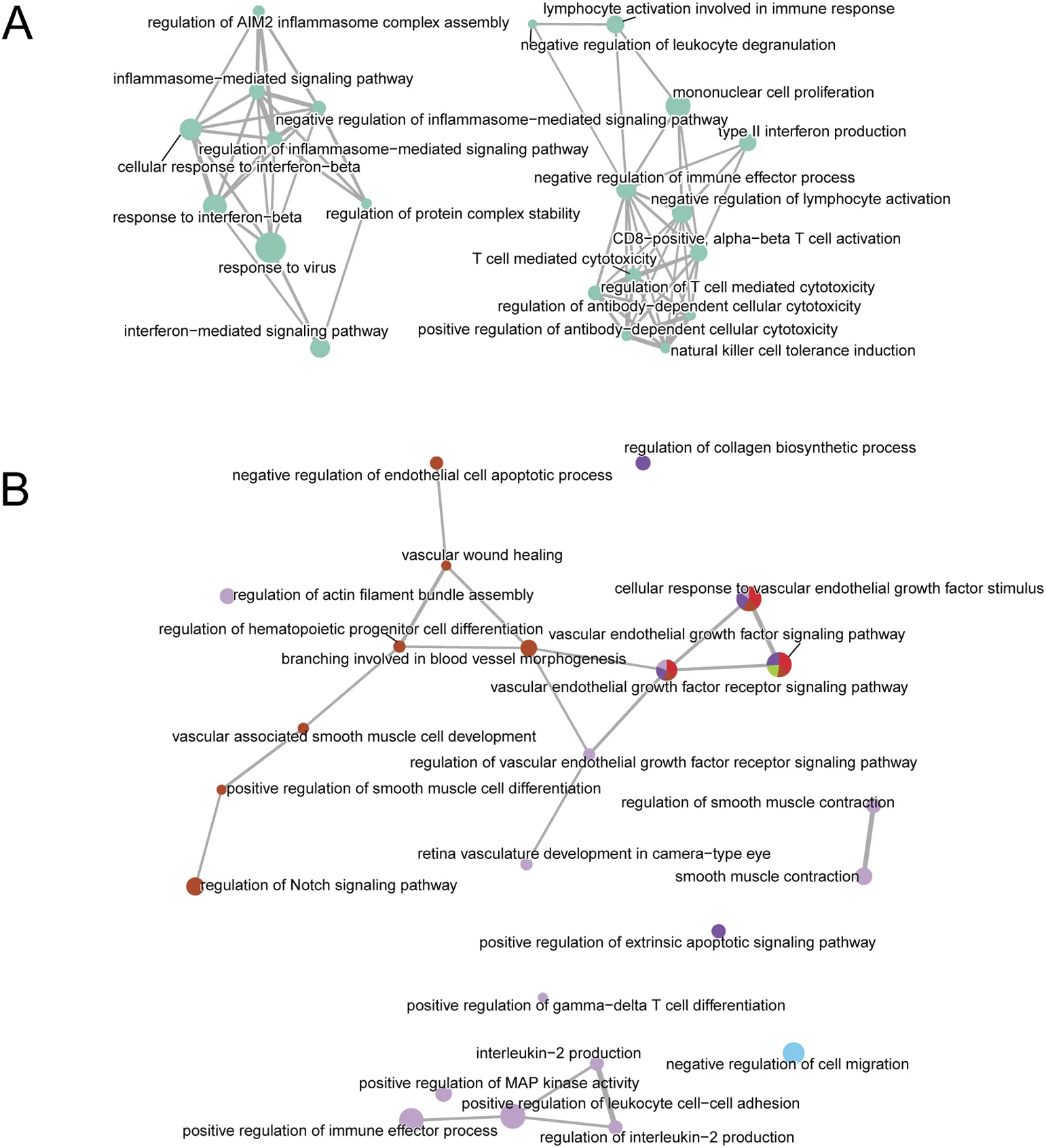


**Figure S10:**