# Supplementary material for: Natural Asp-tRNA synthetase fragment targets myofibroblasts to abrogate tissue fibrosis
Source: J Biol Chem. 2026 Sep 30;302(11):113586. doi: 10.1016/j.jbc.2026.113586 (PMC13755475; doi:10.1016/j.jbc.2026.113586)
Supplement: Supplementary file 1 [file mmc1.pdf]

A

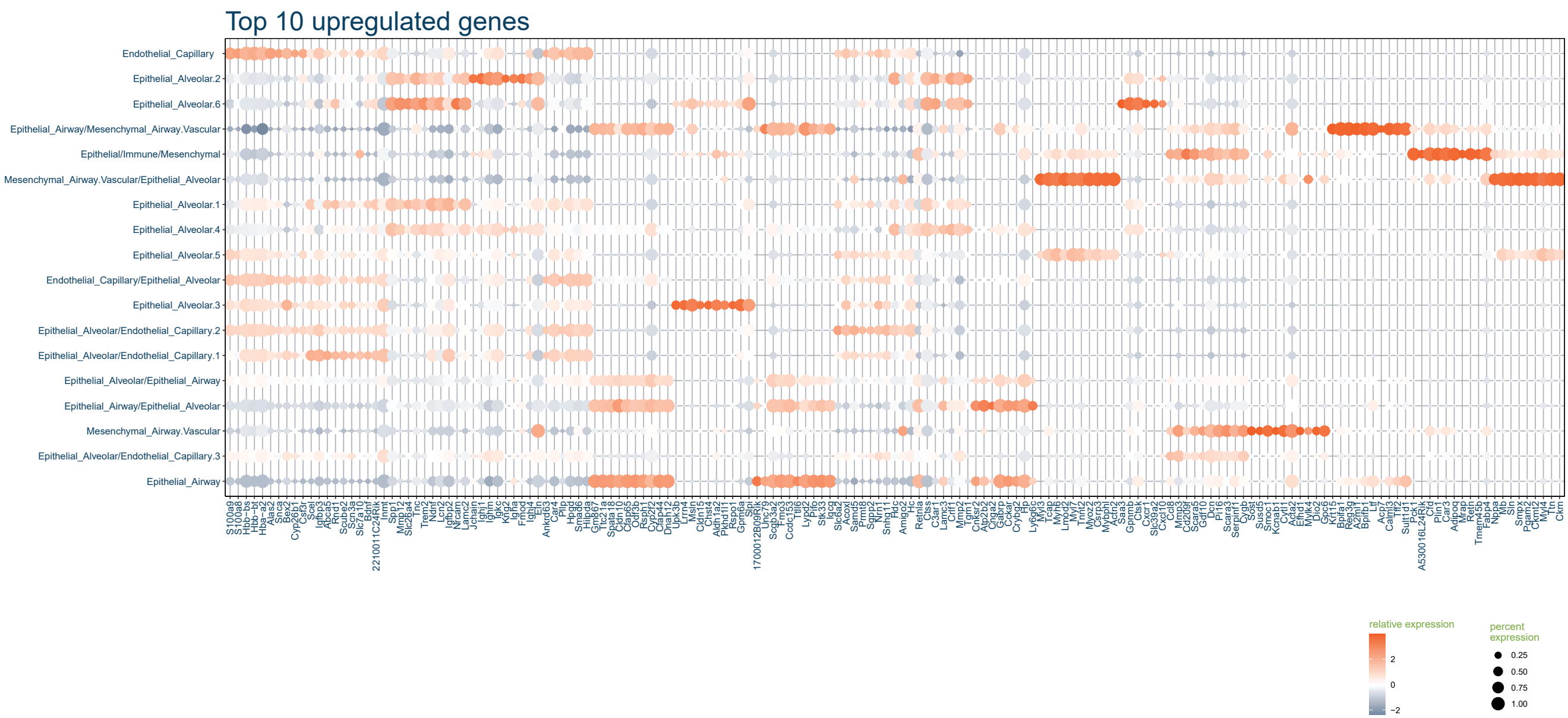

Top 10 upregulated genes
Endothelial_Capillary
Epithelial_Alveolar.2
Epithelial_Alveolar.6
Epithelial_Airway/Mesenchymal_Airway.Vascular
Epithelial/Immune/Mesenchymal
Mesenchymal_Airway.Vascular/Epithelial_Alveolar
Epithelial_Alveolar.1
Epithelial_Alveolar.4
Epithelial_Alveolar.5
Endothelial_Capillary/Epithelial_Alveolar
Epithelial_Alveolar.3
Epithelial_Alveolar/Endothelial_Capillary.2
Epithelial_Alveolar/Endothelial_Capillary.1
Epithelial_Alveolar/Epithelial_Airway
Epithelial_Airway/Epithelial_Alveolar
Mesenchymal_Airway.Vascular
Epithelial_Alveolar/Endothelial_Capillary.3
Epithelial_Airway
relative expression
2
0
−2
percent expression
0.25
0.50
0.75
1.00
