# Supplementary material for: Natural Asp-tRNA synthetase fragment targets myofibroblasts to abrogate tissue fibrosis
Source: J Biol Chem. 2026 Sep 30;302(11):113586. doi: 10.1016/j.jbc.2026.113586 (PMC13755475; doi:10.1016/j.jbc.2026.113586)
Supplement: Supplementary file 2 [file mmc2.docx]

**Supporting Information 1. Proteolysis of full length DARS by PMN elastase and MMP-12. (A)** Full length DARS was incubated with increasing concentrations of PMN elastase and MMP-12 to reveal the proteolytic product termed DARS-N1. Mass spectrometry analysis determined the N-terminal proteolytic fragment of DARS encompasses amino acids 1-154.

**Supporting Information 2. LTBP-1 is identified as a binding partner of ATYR0101.** **(A)** A mass spectrometry-based receptor screen employing a ligand-receptor capture technology identified LTBP-1 as a binding partner of ATYR0101. LTBP-1 peptide sequences identified were highlighted in LTBP-1 AlphaFold structure (entry Q14766). **(B)** WI-38 cells were treated with TGF-β for 6 days to allow for ECM deposition. Cells were removed and ECM was stained with **DARS-N1** or a control protein (green), collagen VI (orange), and LTBP-1 (red).

**Supporting Information 3. Structure, serum exposure and purified protein profile of ATYR0101. (A)** Model of ATYR0101 based on cryoEM density map of the protein (4.96 Å-resolution; PDB ID 21AA, Extended PDB ID pdb_000021AA, EMD-67441). The hIgG Fc and linker portions are labeled in yellow and the DARS-N1 in blue. The molecule forms a dimer due to the Fc moiety. Mutations to improve stability are highlighted in red for R81C and N86C and green for C130S. **(B)** Mice received a single dose of 1 mg/kg DARS-N1 or ATYR0101 via systemic delivery. Pharmacokinetic profiles indicate the levels of drug within serum over the course of one week. **(C)** DARS-N1 (top) and ATYR0101 (bottom) were subjected to SDS-PAGE (left panel) or size-exclusion chromatography (SEC, right panel) analysis for assessing protein purity and homogeneity. Samples were prepared in non-reducing conditions (NR) or reducing conditions (R) as indicated in the SDS-PAGE.

**Supporting Information 4. ATYR0101 binds to C-terminal LTBP-1 fragments in all species tested, including human, mouse, rat and cynomolgus monkey. (A)** Domain organization of full-length LTBP-1S and the constructs used in this study. All proteins were expressed in mammalian cells with FLAG-His tags. **(B)** AlphaLISA confirmed the direct protein-protein binding between ATYR0101 and human LTBP-1 fragments. **(C)** LTBP-1 fragments (position 1006-1185 in purple and position 1186-1395 in orange) were highlighted in LTBP-1 AlphaFold structure. **(D)** Direct protein-protein interaction between ATYR0101 and human (black), mouse (blue), rat (red) and cynomolgus monkey (green) LTBP-1 fragments with position 1006-1395 were confirmed in AlphaLISA.

**Supporting Information 5. ATYR0101 does not induce apoptosis in lung epithelium cells.** Lung epithelial cells, A549, were seeded overnight before treating with ATYR0101 or control protein without **(A)** or with 10ng/mL of TGF-β **(B)** to induce epithelial-to-mesenchymal transition of A549 cells. After 2 days of treatment, caspase-3/7 activities were measured and quantified using Caspase-Glo® 3/7 Assay. Staurosporine (5µM) serves as a positive control. (n = 2 independent studies). Data are presented as mean ± SEM.

**Supporting Information 6. ATYR0101 induced myofibroblast apoptosis is blocked by TGF-β inhibitor treatment.** TGF-β differentiated primary IPF myofibroblasts **(A)** or WI-38 myofibroblasts **(B)** were treated with ATYR0101 or control protein (1µM) in the presence or absence of TGF-β receptor inhibitor SB525334 (3µM). Kinetic plots of annexin V signals are measured through live cell imaging. Data are shown as mean ± SEM. n = 3 independent studies. Scale bars are 400μm.

**Supporting Information 7. ATYR0101 is detectable in both serum and BALF and therapeutic ATYR0101 treatment shows fibrosis reduction potential. (A-B)** ATYR0101 is detected in the serum and BALF of bleomycin-induced mice. Data are shown as a mean ± SEM. n = 5 biological replicates per group. **(C)** Duplex immunofluorescence analysis shows that ATYR0101 colocalizes with LTBP-1 in the lungs of bleomycin challenged mice.

**Supporting Information 8. Gene markers of the cell populations in the bleomycin model of lung fibrosis. (A)** Dot plot showing the top ten upregulated genes in each of the 18 cell populations.

**Supporting Information 9. Features of the cell populations in the bleomycin model of lung fibrosis.** **(A)** Bar plot identifying the proportions of cell lineages that comprise each of the cell populations. **(B)** Heat map comparing the activity of key pathways associated with inflammation in the cell populations and their respective phenotypes. Hierarchical clustering reveals the existence of four groups of cell populations according to transcriptomic pathway activity. Group 1: TGFβ High, inflammatory low; Group 2: TGFβ low, inflammatory low; Group 3: TGFβ medium/low, inflammatory medium/low; Group 4: TGFβ medium/high, inflammatory medium/high. **(C-D)** Gene set enrichment map plots showing biological processes enriched in the cell populations of the fibrotic phenotype.

**Supporting Information 10. Pathway enrichment analysis of the nintedanib-specific and MOA-specific phenotypes.** Gene set enrichment map plots showing biological processes enriched in the cell populations of the nintedanib **(A)** and ATYR0101 **(B)** treatment groups.

**Supporting Information 11. ATYR0101 reduces fibrosis in the UUO model of kidney fibrosis. (A-C)** LTBP-1 expression via IHC in normal healthy mouse kidneys (A,C) as well as fibrotic kidney from the UUO kidney fibrosis model 7 days post-induction (B,C). Data are shown as mean ± SEM. n = 2 (naïve) n = 3 (fibrotic) biological replicates. Scale bars are 2mm (top panels), 200µm (bottom left panel) or 100μm (bottom right panel). **(D)** The UUO kidney fibrosis efficacy study; representative MTC-stained kidney images for each treatment group are shown. Scale bars represent 200µm. **(E-G)** Kidney weights, cortical collagen content, and pathologist scoring for degree of fibrosis for naïve mice and mice treated with either PBS or ATYR0101. Data are shown as a mean ± SEM. n = 5 biological replicates per group for all groups. ***P<0.001, ***P<0.0001 by ordinary one-way ANOVA.
